# *Kombucha* inoculated fermentation reshapes microbial ecology and flavour metabolism in Yunnan Arabica coffee

**DOI:** 10.1038/s41538-026-00852-1

**Published:** 2026-04-17

**Authors:** Shengjie Duan, Jinya Dong, Yuanfeng Chen, Lihui Yu, Shan Liu, Rongxian Yu, Zezhu Du, Yan Shen, Xiuli Lu, Jianyang Fu, Ruijuan Yang, Chongye Fang

**Affiliations:** 1 College of Food Science and Technology, Yunnan Agricultural University, Kunming, China; 2 Jiangnan University School of Food Science and Technology, Wuxi, 214122 China; 3 Yunnan Research Center for Advanced Tea Processing, Kunming, China; 4 Key Laboratory of Development and Utilization of Food and Medicinal Resources, Ministry of Education, Yunnan Agricultural University, Kunming, 650201 China

**Keywords:** Biotechnology, Microbiology, Plant sciences

## Abstract

This study evaluates the flavor-enhancing effects of *kombucha*-inoculated fermentation on *Coffea arabica L*. and uncovers regulatory mechanisms across microbial succession, physicochemical shifts, amino acid remodeling, and volatile formation. Controlled fermentations using *kombucha* symbiotic consortium for 144 h was comparedwith spontaneous fermentation. At endpoint, bacterial richness in the KT group was 34% higher compared to the CK group. The KT group exhibited a significantly lower pH (4.21) than the CK group (4.95). *Komagataeibacter* and *Zygosaccharomyces* were enriched 2–6 fold, while *Enterobacter* and *Aspergillus* were suppressed. *Kombucha* coffee showed lower *pH*, titratable acidity increased by 64%, and reducing sugars decreased by 43%. Sweet-taste FAAs increased and bitter FAAs decreased, correlating with floral–fruity esters (*r* ≥ 0.74). Volatiles such as *phenylethyl alcohol* (42%), *phenethyl acetate* (200%), and *ethyl isovalerate* (89%), while off-flavor acids and smoky phenols decreased. Sensory scores improved in floral, fruity, and sweet attributes. Multi-omics linked dominant taxa to upregulated pathways (*ester biosynthesis, aromatic amino acid degradation, Maillard products*) and key functional genes. These results establish *kombucha* Inoculated Fermentation as a reproducible, mechanism-based strategy for targeted flavor optimization in speciality coffee and other high-value agricultural products.

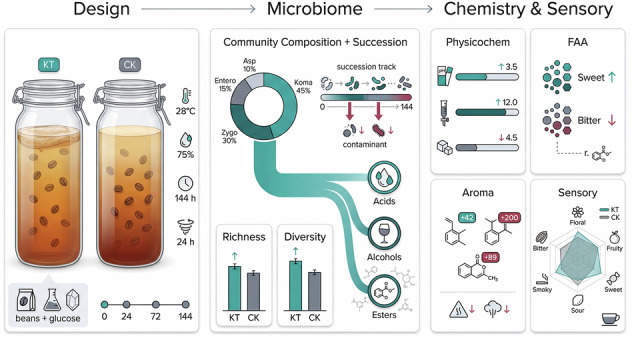

## Introduction

Coffee flavor is fundamentally dictated by its geographical origin, often referred to as ‘terroir’. While regions like Ethiopia and Colombia are renowned for their distinct floral and citrus notes driven by high altitude and specific processing, *Coffea arabica L*. is among the world’s most heavily consumed indulgence beverages^1^, and its quality is tightly coupled to market price. Grown at 1200–1800 m in Yunnan’s highland climate^2^, the region’s *Coffea arabica L*. exhibits vibrant fruit acidity (*peaberry*) and considerable floral potential. Nevertheless, conventional dry or washed processing often yields beans with an unbalanced acid–sweet ratio, a pronounced bitter–astringent aftertaste, and limited aromatic complexity^3^. In recent years, Controlled fermentation is most commonly applied during the wet processing stage, replacing spontaneous microbial activity with specific starter cultures to ensure consistent mucilage removal and flavor formation. controlled fermentation has emerged as a promising route to remedy these limitation^4^. Monoculture inoculation with yeasts such as *Saccharomyces cerevisiae* or lactic acid bacteria such as *Lactiplantibacillus plantarum* can depolymerize pectins, chlorogenic acids, and proteins, boosting short-chain esters and higher alcohols and thereby modestly improving flavor^5^. Yet the metabolic repertoire of single strains is inherently narrow; the resulting aroma spectrum remains restricted, and concerted modulation of acidity and flavor remains elusive^6^.

*Kombucha* is a tea-based fermentation system governed by a symbiosis of acetic-acid bacteria and yeasts^7^. During *Kombucha* ferementation, *Komagataeibacter spp*. produce acetic, gluconic, and other organic acids^8^, whereas *Zygosaccharomyces* and *Brettanomyces spp*. generate a rich spectrum of alcohols and esters, bequeathing the beverage its crisp sweet–sour balance and floral–fruity bouquet^9^. Recent research demonstrates that constructing a tailor-made symbiotic microbial consortium for *kombucha* fermentation can significantly enhance the antioxidant capacity and sensory complexity of polyphenol-rich substrates like apple juice, driven by the targeted modulation of functional metabolites and volatile esters^10^. These attributes position the *kombucha* consortium as a promising composite starter for the simultaneous enhancement of flavor and safety. Traditionally, Yunnan Arabica coffee is processed via spontaneous wet fermentation, a process driven by indigenous microbes such as *Saccharomyces cerevisiae, Lactiplantibacillus plantarum*, and *Bacillus spp*. which degrade mucilage and initiate flavor precursor formation. However, the inconsistency of these wild populations often leads to unpredictable cup quality and flavor defects. *Kombucha* was specifically chosen as the inoculant because its symbiotic community *(SCOBY)* uniquely combines the organic acid production of *Komagataeibacter* with the ester-synthesizing capability of yeasts. This dual metabolic mechanism is theoretically ideal for systematically correcting the insufficient acidity and lack of aroma complexity often found in traditionally processed Yunnan coffee.

Green beans of *Coffea arabica L*. are enriched with soluble sugars, free amino acids, and phenolic acids^11^, affording the *kombucha* consortium a diverse palette of carbon and nitrogen substrates^12^. Within a temperature- and humidity-controlled milieu, yeasts transmute these saccharides into alcohols and esters, amplifying floral–fruity nuances and sweetness^13^, while organic acids secreted by *Komagataeibacter* adjust pH, impart a brisk acidity, and may, through non-enzymatic reactions, diminish precursors of bitterness and astringency^14^. The consortium’s low pH and polyphenol-derived metabolites also curtail moulds and potential toxin-producing microorganisms, thus reinforcing safety^15^. Nevertheless, a comprehensive elucidation of the microbial succession, physicochemical trajectories, and key aroma-forming mechanisms engendered by *kombucha* as a starter in coffee processing not yet been fully elucidated.

In a previous study, the primary objective of this study was to *Coffea arabica L*. was subjected to *kombucha*-inoculated fermentation and spontaneous fermentation controls^16^. Specifically, we aimed to: (i) track the microbial community shifts, Microbial succession was tracked by high-throughput sequencing, while physicochemical indices—including soluble solids, reducing sugars, total acidity, and free amino acids—were monitored in parallel^17^; (ii) monitor physicochemical changes, Volatile compounds were qualitatively and quantitatively profiled by *HS–SPME–GC–MS*. Odor contributions were assessed via *OAV(Odor Activity Value)/ROAV*, and discriminatory patterns were interrogated using *OPLS-DA* and Pearson correlation analysis to elucidate the regulatory impact and underlying mechanisms of *kombucha* composite fermentation on flavor quality; and (iii) elucidate the mechanisms of volatile flavor enhancement. The findings are intended to furnish a scientific foundation for the high-value processing of Yunnan coffee and to extend the application horizon of *kombucha* in the deep fermentation of specialty agricultural products.

## Results and discussion

### Microbial community structure in coffee after fermentation

#### *α-*Diversity

Following rigorous quality control of 24 samples, a total of 1,486,312 high-quality bacterial sequences were obtained (mean per sample: 62,000 ± 2915), along with 1,153,479 high-quality fungal sequences (mean per sample: 48,062 ± 3107). As shown in Table 1, Good’s Coverage indices ranged from 0.973 to 0.988, indicating that the sequencing depth was sufficient to comprehensively capture community diversity^18^.

**Table 1:** Sequencing statistics for bacterial and fungal communities during kombucha-inoculated (KT) and natural (CK) fermentations of Yunnan Arabica coffee beans

Sample (treatment – time)	Valid bacterial reads (×10³)	Valid fungal reads (×10³)	Good’s coverage (bacteria)	Good’s coverage (fungi)
CK – 0 h	61.41 ± 2.24	47.92 ± 1.61	0.98 ± 0.002	0.97 ± 0.003
KT – 0 h	62.22 ± 2.54	48.16 ± 1.71	0.98 ± 0.002	0.97 ± 0.002
CK – 24 h	62.31 ± 2.67	48.08 ± 2.12	0.98 ± 0.002	0.98 ± 0.002
KT – 24 h	63.85 ± 2.79	48.48 ± 2.03	0.98 ± 0.002	0.97 ± 0.002
CK – 72 h	61.76 ± 2.95	48.37 ± 2.33	0.98 ± 0.001	0.98 ± 0.002
KT – 72 h	63.01 ± 2.41	48.01 ± 2.21	0.98 ± 0.001	0.98 ± 0.002
CK – 144 h	62.52 ± 3.01	48.75 ± 2.45	0.99 ± 0.001	0.98 ± 0.002
KT – 144 h	62.01 ± 2.53	48.83 ± 2.24	0.99 ± 0.001	0.98 ± 0.002

*CK* natural fermentation control, *KT* kombucha-inoculated fermentation. (mean ± SD, *n* = 3), Good’s coverage (dimensionless).

**Fig. 1:**
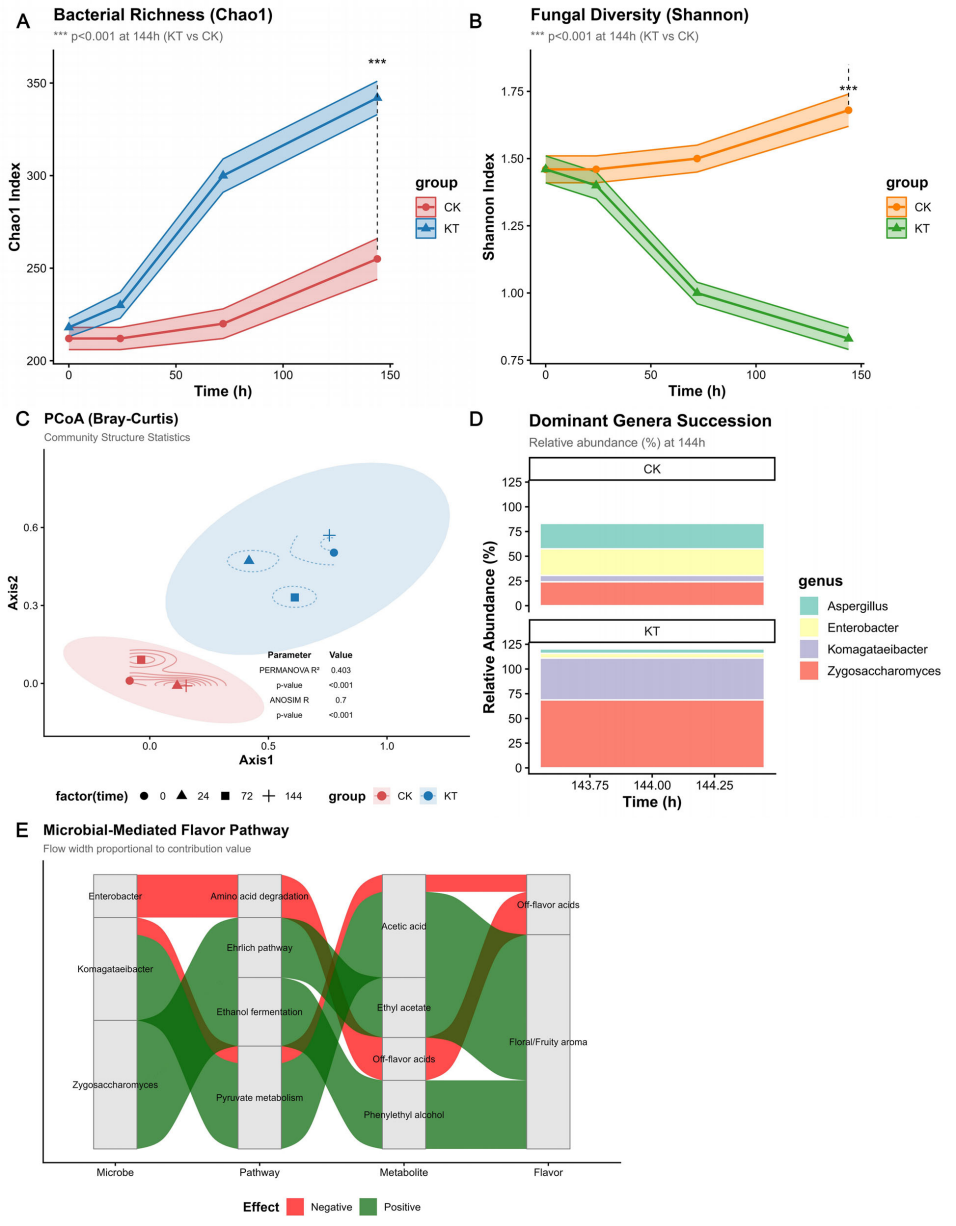
Kombucha inoculated fermentation reshapes microbial ecology and flavor metabolism in Yunnan Arabica Coffee. Overview of how Kombucha-inoculated (KT) fermentation alters microbial community structure and flavor-related metabolism in Yunnan Arabica coffee compared to spontaneous control (CK). Each condition and time point was assessed with three biological replicates (*n* = 3). **A** Bacterial richness (Chao1 index): KT showed a significant increase vs CK at 144 h (***p < 0.001). **B** Fungal diversity (Shannon index): KT exhibited significantly reduced diversity at 144 h (***p < 0.001), indicating selective suppression. **C** Beta diversity (PCoA, Bray-Curtis): Clear separation between KT and CK over time; PERMANOVA and ANOSIM confirm significant differences (*p* < 0.001). **D** Dominant genera succession: At 144 h, KT enriched beneficial taxa (e.g., Komagataeibacter) and suppressed undesirable ones (***p < 0.001, **p < 0.01). **E** Microbe–Pathway–Flavor linkage: Sankey diagram shows microbial contributions to metabolic pathways and flavor attributes, with green indicating positive and red indicating negative effects.

*Kombucha*-inoculated fermentation significantly modulated the alpha-diversity, which represents the richness and evenness of microbial species within a single sample of both bacterial and fungal communities. The bacterial Chao1 richness index in the *Kombucha*-treated (KT) group consistently exceeded that of the spontaneous control (CK) group throughout the fermentation process, with a pronounced and statistically significant difference observed at 144 h (Fig. 1A). Specifically, at the initial stage (0 h), no significant difference was observed between CK and KT groups (212 ± 6 vs. 218 ± 5; *p* > 0.05). However, after 144 h of fermentation, the Chao1 index in the KT group surged to 342 ± 9, markedly higher than that of the CK group at 255 ± 11 (*p* < 0.001). Conversely, The fungal Shannon diversity index in the KT group exhibited a downward trend over time and was significantly lower than that of the CK group at 144 h (declining from 1.46 ± 0.05 to 0.83 ± 0.04 in KT, compared to the CK group’s 1.68 ± 0.06; *p* < 0.001) (Fig. 1B). These findings indicate that *Kombucha* inoculation not only enhances bacterial richness but also effectively suppresses fungal diversity, thereby reshaping the microbial environment^19^.

#### *β-*Diversity

An in-depth examination of beta-diversity shifts further elucidated the profound impact of *Kombucha* inoculation on microbial community structure. The Principal Coordinates Analysis (PCoA) plot, based on Bray–Curtis distances, presents the distinct temporal succession of bacterial communities (Fig. 1C). The PCoA trajectory shows an initial overlap between CK and KT groups at 0 h, followed by a gradual divergence after 24 h, culminating in maximal separation at 144 h. Transparent 95% confidence ellipses clearly delineate the spatial distribution and clustering of the communities for each group, with geom_density_2d contours highlighting the regions of highest sample density. Statistical analyses confirmed substantial intergroup differences in community structure: PERMANOVA revealed an R² of 0.403 with a *p*-value of 0.001, while ANOSIM likewise indicated a strong divergence between groups with an R-statistic of 0.7 and a *p*-value of 0.001 (Fig. 1C, inset table). This collective evidence underscores the decisive role of *Kombucha* inoculation in reshaping microbial community architecture throughout the fermentation process^20^. Furthermore, a detailed Bray–Curtis dissimilarity heatmap (Supplementary Fig. 1) visually conveys the pairwise distance matrix among all samples, revealing that KT group samples exhibited pronounced clustering in the later stages, with dissimilarities to CK group samples markedly exceeding intragroup variation.

### Bacterial–fungal succession and functional associations

The dynamic evolution and interrelationships of key microbial genera during fermentation were further elucidated. The relative abundances of dominant taxa revealed that in the KT group, *Komagataeibacter* and *Zygosaccharomyces* exhibited a marked increase throughout fermentation, ultimately prevailing at 144 h (*Komagataeibacter*: 42.3 ± 2.5%, significantly surpassing CK’s 6.5 ± 0.9%, *p* < 0.001; *Zygosaccharomyces*: stably maintained at 68.7 ± 3.3% in KT, far exceeding CK’s 24.1 ± 2.7%, *p* < 0.001) (Fig. 1D). Conversely, *Enterobacter* and *Aspergillus* displayed higher abundances in CK, yet were conspicuously suppressed in KT (*Enterobacter* declined from 21.4 ± 2.2% to 4.8 ± 0.7% in KT, while CK remained at 26.9 ± 2.4%, *p* < 0.001; *Aspergillus* consistently remained below 5% in KT but expanded to 25.7 ± 2.0% in CK, *p* < 0.01). The dominance of *Komagataeibacter* over *Acetobacter* in this study aligns with recent taxonomic reclassifications and reflects its superior ecological fitness in the coffee-sucrose environment. *Komagataeibacter* is known for its high efficiency in organic acid biosynthesis, which likely outcompeted other acetic acid bacteria during the 144 h fermentation.

To complement the community succession analysis, Fig. 1E provides an integrated view of the microbe–pathway–metabolite axis, illustrating how specific genera drive the metabolic flux toward desirable flavor compounds. This multi-layered alluvial diagram vividly maps the influence of key genera (*Komagataeibacter, Zygosaccharomyces, Enterobacter*) through specific functional pathways (e.g., *pyruvate metabolism, ethanol fermentation, aromatic amino acid degradation*) on the production of flavor metabolites (e.g., *acetic acid, higher alcohols, esters*, and *bitter amino acids*). The thickness of the connecting lines conveys the intensity of metabolic flux, and color coding distinguishes positive (green) from negative (red) contributions, underscoring the multilayered regulatory role of the *kombucha* consortium in flavor development. For instance, *Komagataeibacter* promotes acetic acid production via pyruvate metabolism, while *Zygosaccharomyces* drives ethanol fermentation and aromatic amino acid degradation, generating higher alcohols and esters (positive contributions). In parallel, the inhibitory effect of *Enterobacter* on aromatic amino acid degradation is diminished in KT, reducing undesirable flavor compounds such as bitter amino acids^21^. This synergy culminates in an “enhanced flavor spectrum” by orchestrating precise metabolic control to refine and elevate the coffee flavor profile.

Functional predictions via PICRUSt2, based on 16S rRNA gene sequences, demonstrated that between 72 and 144 h, KT samples exhibited significantly higher abundances (*q* < 0.05) in pathways such as pyruvate metabolism, aromatic amino acid degradation, and ethanol fermentation^22^. Spearman correlation analysis (Benjamini–Hochberg adjustment, *q* < 0.05) further revealed specific associations: *Komagataeibacter* correlated strongly and negatively with *pH* (*r* = −0.85) while showing a robust positive association with acetic acid (*r* = 0.83); *Zygosaccharomyces* was positively correlated with ethyl acetate (*r* = 0.79) and phenethyl alcohol (*r* = 0.74); *Enterobacter* exhibited a negative correlation with acetic acid (*r* = −0.72). The differential pathway enrichment heatmap (Supplementary Fig. 1) distinctly presents the Log₂FC and p-values for multiple functional pathways between KT and CK, with blue-shaded regions (e.g., *ethanol fermentation, aromatic amino acid degradation, organic acid biosynthesis, pyruvate metabolism*) denoting pathways significantly upregulated in KT, whereas red-shaded regions (e.g., *glycolysis, lipid metabolism*) reflect pathways upregulated in CK^23^.

### Physicochemical attributes of fermented coffee

**Fig. 2:**
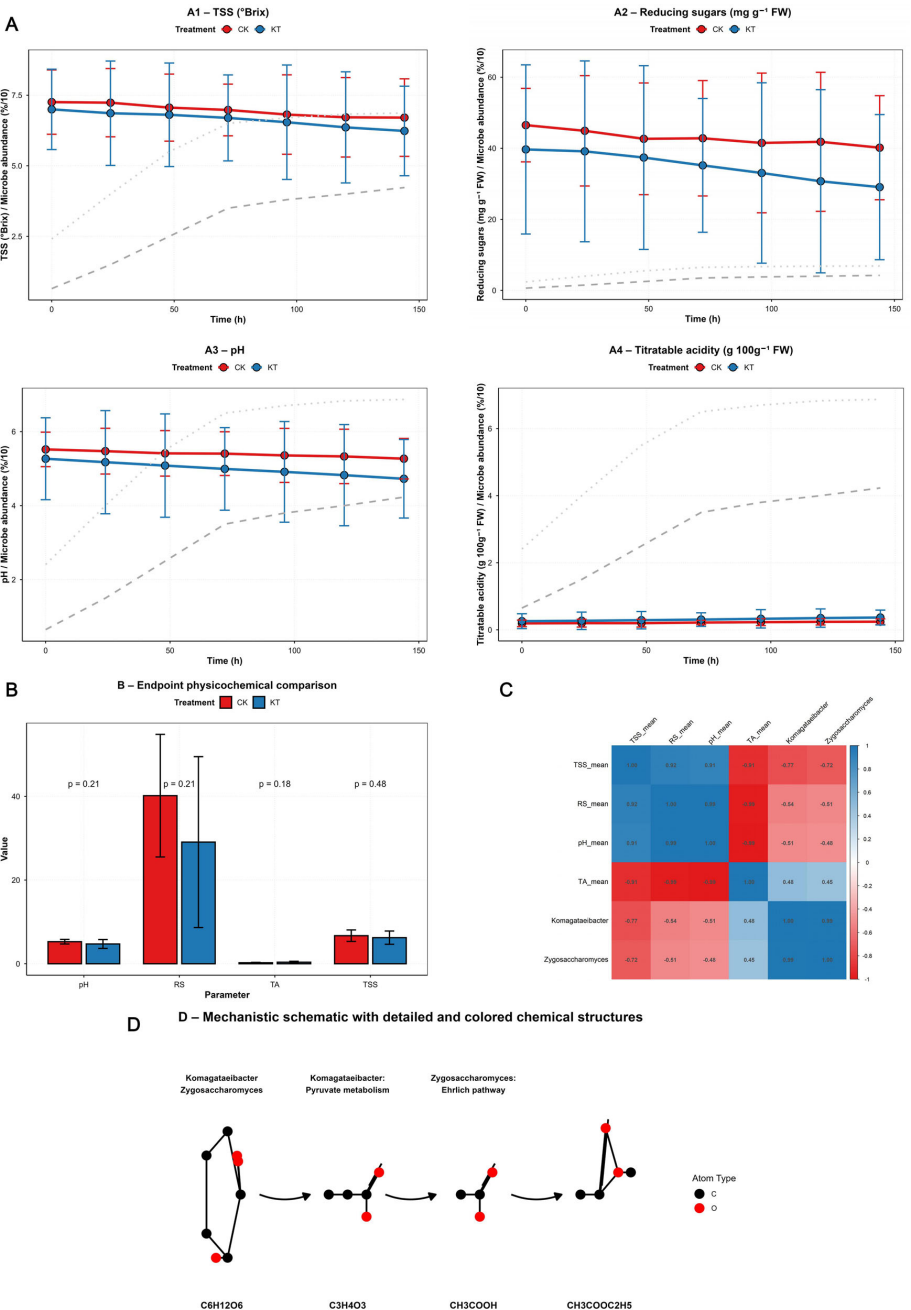
Physicochemical dynamics and metabolic mechanisms during kombucha-inoculated fermentation of Yunnan arabica coffee. **A1**–**A4** Physicochemical parameters and microbial abundance during fermentation. Time-course of TSS (°Brix), reducing sugars (RS), pH, and titratable acidity (TA) in kombucha-inoculated (KT, blue) vs control (CK, red) over 144 h (*n* = 3, mean ± SD), with overlaid relative abundance of *Komagataeibacter* (dashed) and *Zygosaccharomyces* (dotted). Significant differences indicated (t-test). **B** Endpoint comparison. Bar plots of pH, RS, TA, and TSS at 144 h (p-values from t-test). **C** Correlation heatmap. Pearson’s r between mean physicochemical traits and dominant genera abundance (blue = positive, red = negative). **D** Mechanistic schematic. Glucose (C6H12O6) → pyruvate (C3H4O3) → acetic acid (CH3COOH) via *Komagataeibacter*; alcohols via *Zygosaccharomyces*; ester formation (e.g., ethyl acetate CH3COOC2H5) from acid–alcohol reaction.

During *Kombucha-inoculated fermentation* (KT), the physicochemical properties of Yunnan Arabica coffee beans underwent pronounced and dynamic transformations, standing in sharp contrast to the natural fermentation control group (CK), with these shifts—driven by microbial metabolism—profoundly influencing flavor development (Fig. 2A–D; Table 2).

**Table 2:** Physicochemical parameters of Yunnan Arabica coffee during fermentation with (KT) and without (CK) kombucha inoculation

Treatment	Time (h)	TSS (°Brix)	Reducing sugars (mg g⁻¹ FW)	pH	Titratable acidity^a^ (mg g⁻¹ FW)
CK	0	7.83 ± 0.21 aA	52.31 ± 2.41 aA	5.80 ± 0.03 aA	0.15 ± 0.01 aA
	24	7.24 ± 0.11 aB	44.12 ± 1.73 bB	5.44 ± 0.04 bB	0.21 ± 0.02 bB
	72	6.51 ± 0.32 bC	37.31 ± 2.63 cC	5.18 ± 0.06 cC	0.25 ± 0.02 cC
	144	6.01 ± 0.14 cD	34.03 ± 2.02 cC	4.95 ± 0.05 dD	0.28 ± 0.01 cC
KT	0	7.92 ± 0.11 aA	51.84 ± 2.13 aA	5.79 ± 0.02 aA	0.15 ± 0.01 aA
	24	6.91 ± 0.23 bB	36.74 ± 1.91 bB	4.92 ± 0.04 cC	0.30 ± 0.03 dD
	72	5.83 ± 0.34 cC	24.81 ± 2.31 dD	4.54 ± 0.06 eE	0.38 ± 0.02 eE
	144	5.41 ± 0.24 dD	19.41 ± 1.44 eE	4.21 ± 0.03 fF	0.46 ± 0.02 fF

Different lowercase letters in the same column indicate significant differences among time points within the same treatment; different uppercase letters indicate significant differences between treatments at the same time point (Tukey, *p* < 0.05) (mean ± SD, *n* = 3).

^a^Expressed as acetic acid equivalents titrated with 0.1 mol L⁻¹ NaOH to pH 8.1.

Continuous monitoring of total soluble solids (TSS, Fig. 2A1), reducing sugars (Fig. 2A2), pH (Fig. 2A3), and total titratable acidity (TA, Fig. 2A4) over 144 h revealed that the *kombucha* consortium displayed markedly greater efficiency in transforming the coffee substrate than natural fermentation. At the initial stage (0 h), TSS and reducing sugar contents did not differ significantly between groups. However, as fermentation progressed, TSS in KT declined steadily, reaching 5.4 ± 0.2 °Brix at 144 h—significantly lower than CK’s 6.0 ± 0.1 °Brix (*p* < 0.05). Similarly, reducing sugars in KT were consumed more rapidly, plummeting to 19.4 ± 1.4 mg g^−1^FW at 144 h, far below CK’s 34.0 ± 2.0 mg g^−1^FW (*p* < 0.01). This accelerated carbohydrate depletion mirrored the synchronous, rapid rise in the abundances of *Komagataeibacter* and *Zygosaccharomyces* in KT (Fig. 2A1–A4, gray dashed and dotted lines), underscoring the consortium’s potent capacity for carbon utilization^24^.

Carbohydrate consumption was accompanied by pronounced acidification^25^. The *pH* in KT dropped sharply from an initial 5.79 ± 0.02 to 4.21 ± 0.03 at 144 h, substantially lower than CK’s 4.95 ± 0.05, with significant divergence emerging as early as 24 h (*p* < 0.001). Correspondingly, TA in KT accumulated steadily, reaching 0.46 ± 0.02 g 100 g^−1^FW at 144 h—significantly exceeding CK’s 0.28 ± 0.01 g 100 g^−1^FW (*p* < 0.001). The *pH* decline and TA elevation reflected the vigorous acidogenic metabolism of *Komagataeibacter*, imparting a bright acidity to the coffee while creating an inhibitory environment for spoilage microorganisms^26^.

Endpoint comparisons at 144 h (Fig. 2B) further highlighted KT’s physicochemical advantages: significantly lower *pH* (*p* < 0.001), markedly higher TA (*p* < 0.001), and substantially reduced reducing sugars (*p* < 0.01) relative to CK. Together, these parameters form the distinctive physicochemical foundation of *kombucha*-fermented coffee.

To elucidate the microbial drivers underlying these changes, correlations between physicochemical indices and dominant genera were examined (Fig. 2C). *Komagataeibacter* abundance exhibited a strong negative correlation with *pH* (*r* = –0.91) and a near-perfect positive correlation with TA (*r* = 0.99), directly confirming its central role as the principal acetic acid producer in coffee acidification. Both *Komagataeibacter* and *Zygosaccharomyces* correlated negatively with TSS (*r* = –0.77 and *r* = –0.72) and reducing sugars (*r* = –0.54 and *r* = –0.51), indicating their joint participation in carbohydrate depletion. These robust associations provide compelling evidence that the *kombucha* microbiota actively reshapes the physicochemical profile of coffee during fermentation^27^.

The potential metabolic mechanisms by which the *kombucha* consortium modulates physicochemical properties (Fig. 2D). In the early fermentation phase, glucose (C_6_H_12_O_6_) abundant in coffee beans served as the primary substrate. Through the synergistic activity of *Komagataeibacter* and *Zygosaccharomyces*, glucose underwent catabolism. *Komagataeibacter* prominently activated the pyruvate metabolism pathway, converting glucose into pyruvate (C_3_H_4_O_3_) and subsequently oxidizing it to acetic acid (CH_3_COOH)^28^. The accumulation of acetic acid was the principal driver of *pH* reduction and TA elevation^29^. Concurrently, *Zygosaccharomyces* predominantly utilized the Ehrlich pathway, metabolizing amino acids and sugar intermediates to generate higher alcohols, including ethanol. These microbially derived acetic acid and ethanol molecules subsequently underwent enzymatic or non-enzymatic esterification, yielding key aromatic esters such as ethyl acetate (CH_3_COOC_2_H_5_)^30^.

This intricate cascade of biochemical conversions, orchestrated by microbial metabolism, directly regulated the coffee’s acid–base balance and established the precursor pool for subsequent flavor compound synthesis—ultimately endowing *kombucha*-fermented coffee with its distinctive sensory profile. The final pH of 4.21 in the KT group, though higher than traditional liquid *kombucha*, is attributed to the inherent buffering capacity of coffee bean proteins. Importantly, this pH is significantly below the safety threshold of 4.6, effectively inhibiting the growth of pathogens while maintaining the balanced acidity required for specialty coffee quality. While the reduction in reducing sugars and amino acids in KT might theoretically limit the extent of the Maillard reaction, the remaining levels were sufficient to generate key roast attributes. Moreover, the significant accumulation of organic acids and esters during fermentation likely compensated for the lower sugar content, interacting with Maillard products to create a more complex and balanced flavor profile, avoiding the excessive bitterness or burnt notes sometimes associated with high-sugar roasting.

### Free Amino-Acid Profile of Fermented Coffee

**Fig. 3:**
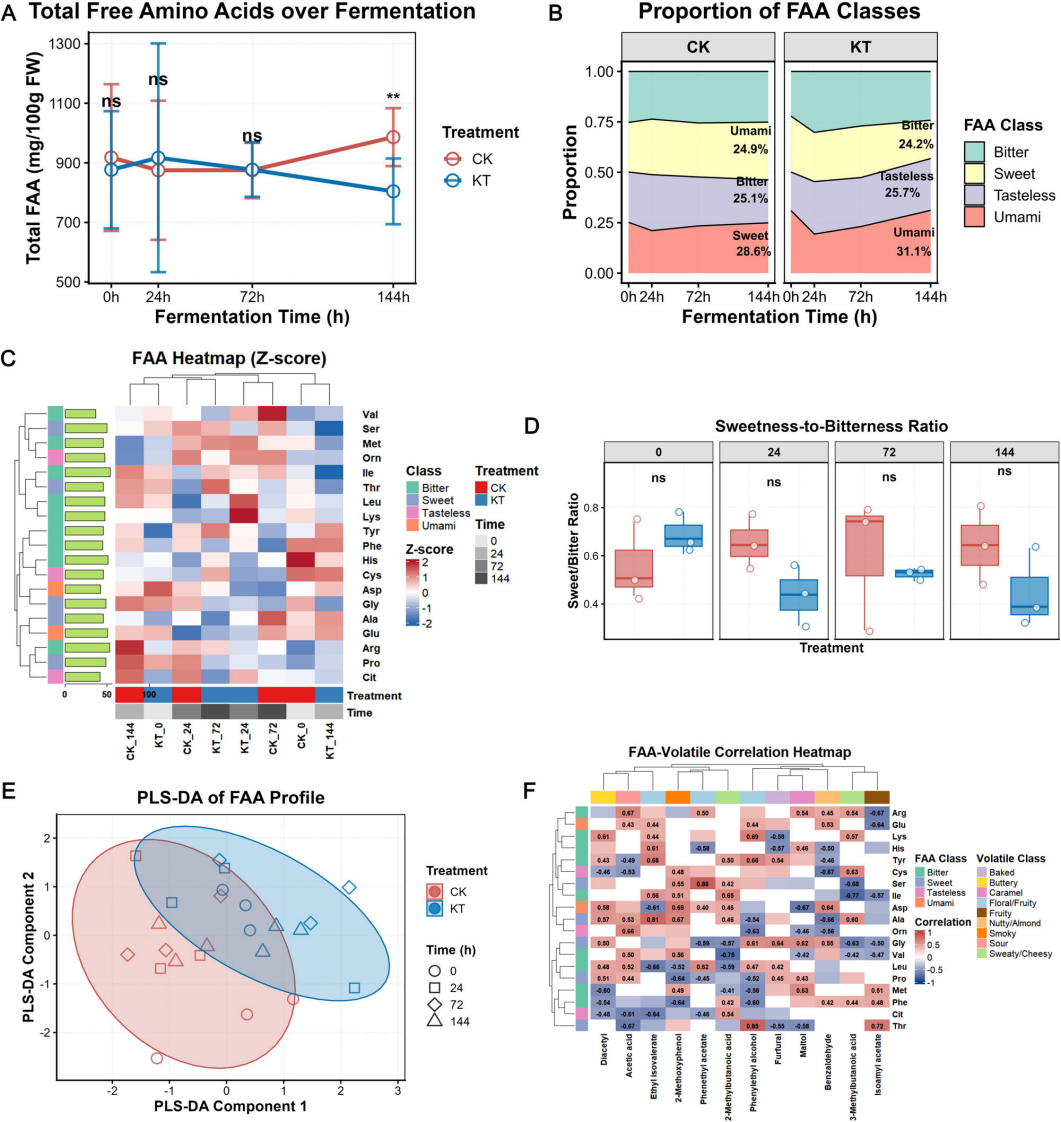
Comprehensive Analysis of Free Amino Acid Profile and Flavor Correlation in Fermented Coffee. Dynamic changes in free amino acid (FAA) profiles and their correlations with key volatile compounds during coffee fermentation (CK: control; KT: kombucha-inoculated). Data are mean ± SD (n = 3). **A** Temporal FAA content (mg 100 g⁻¹ FW) over 144 h; Student’s t-test significance: ns, p ≥ 0.05; **, p < 0.01. **B** FAA functional class distribution (Umami, Sweet, Bitter, Tasteless) in CK and KT; stacked areas show relative proportions, with % values at 144 h. **C** FAA heatmap (Z-score normalized) with hierarchical clustering; left bar denotes functional class; right dots mark significant CK–KT differences at 144 h (p < 0.05). **D** Sweetness-to-Bitterness ratio at 0, 24, 72, 144 h; box plots with t-test results (ns, not significant). **E** PLS-DA score plot showing metabolic separation and temporal trajectories between CK (red) and KT (blue); ellipses indicate 95% confidence regions. **F** Correlation heatmap between FAAs and volatiles; Pearson’s r values (|r| > 0.4) shown; red = positive, blue = negative; clustering by FAA class and volatile category reveals flavor formation links.

During the early stages of fermentation (0 h, 24 h, 72 h), no significant differences (*p* ≥ 0.05) were observed in the total free amino acid (FAA) content between CK and KT treatments (Fig. 3A). However, in the later stage (144 h), the CK group exhibited a significantly higher total FAA content compared with the KT group (*p* < 0.01). This suggests that *Kombucha* inoculation (KT) may either promote the conversion of certain FAAs into other metabolic products or suppress FAA biosynthesis during the terminal phase of fermentation, thereby resulting in a lower net FAA accumulation relative to the control^31^.

The proportional distribution of FAAs across four functional taste categories—umami, sweet, bitter, and tasteless. By the end of fermentation (144 h), the CK group comprised 28.6% sweet FAAs, 25.1% bitter FAAs, and 24.9% umami FAAs, with the proportion of tasteless FAAs not specified in the figure (Fig. 3B). In the KT group, umami FAAs accounted for 31.1%, bitter FAAs for 24.2%, and tasteless FAAs for 25.7%, while the proportion of sweet FAAs was not displayed. These shifts in category proportions imply that *kombucha* fermentation may subtly influence the overall gustatory balance of coffee.

Z-score standardized heatmap of all FAAs across treatments and time points (Fig. 3C). Hierarchical clustering groups FAAs and samples with similar abundance patterns. The annotation bar on the left denotes FAA taste categories (bitter, sweet, tasteless, umami), while the adjacent green bar chart provides average concentration values for each FAA. At the bottom, annotation bars (gray for time, red for CK, blue for KT) clearly identify each sample. Red dots on the right mark FAAs with significant differences between CK and KT at 144 h. Comprehensive concentration data and statistical comparisons for all FAAs are detailed in Table 3.

**Table 3:** Free amino-acid profile of Yunnan Arabica coffee before (0 h) and after fermentation (144 h) with kombucha inoculation (KT) versus control (CK)

Taste class	Amino acid	0 h-CK	0 h-KT	144 h-CK	144 h-KT
Umami	Asp	49.13 ± 1.21 aA	48.81 ± 1.12 aA	63.23 ± 1.82 bA	55.94 ± 1.42 cB
	Glu	76.24 ± 2.03 aA	76.31 ± 1.91 aA	89.54 ± 2.73 bA	77.23 ± 2.23 cB
	Total	125.32 ± 2.81 aA	125.11 ± 2.62 aA	152.71 ± 3.11 bA	133.13 ± 3.01 cB
Sweet	Thr	12.02 ± 0.41 aA	11.83 ± 0.54 aA	9.61 ± 0.31 bB	20.44 ± 0.81 cA
	Ser	18.12 ± 0.61 aA	17.91 ± 0.71 aA	13.11 ± 0.51 bB	16.91 ± 0.64 cA
	Gly	17.11 ± 0.53 aA	16.93 ± 0.53 aA	24.23 ± 0.71 bA	20.51 ± 0.62 cB
	Ala	33.84 ± 1.11 aA	34.02 ± 0.92 aA	47.51 ± 1.43 bA	39.52 ± 1.31 cB
	Pro	15.92 ± 0.72 aA	15.73 ± 0.82 aA	23.63 ± 0.93 bA	29.53 ± 1.11 cA
	Total	96.92 ± 2.21 aA	96.33 ± 2.41 aA	118.04 ± 2.92 bB	127.01 ± 3.33 cA
Bitter	Val	23.72 ± 1.02 aA	23.61 ± 0.94 aA	32.04 ± 0.83 bA	24.82 ± 1.01 cB
	Ile	21.84 ± 0.93 aA	21.91 ± 0.83 aA	30.22 ± 0.72 bA	23.34 ± 1.03 cB
	Leu	45.62 ± 1.61 aA	45.53 ± 1.73 aA	48.94 ± 1.52 aA	42.14 ± 1.43 aB
	Tyr	7.82 ± 0.31 aA	7.92 ± 0.32 aA	11.02 ± 0.41 bA	13.43 ± 0.54 cA
	Phe	18.74 ± 0.72 aA	18.81 ± 0.62 aA	22.12 ± 0.82 bA	22.91 ± 0.94 bA
	His	11.03 ± 0.52 aA	11.14 ± 0.44 aA	15.81 ± 0.64 bA	14.72 ± 0.51 bA
	Arg	3.11 ± 0.23 aA	3.03 ± 0.23 aA	0.82 ± 0.14 bB	5.44 ± 0.34 cA
	Met	10.22 ± 0.41 aA	10.33 ± 0.31 aA	12.12 ± 0.51 bA	11.91 ± 0.52 bA
	Lys	39.91 ± 1.81 aA	40.12 ± 1.74 aA	48.53 ± 2.01 bA	42.14 ± 1.92 cB
	Total	181.81 ± 3.52 aA	182.12 ± 3.12 aA	224.62 ± 4.24 bA	203.43 ± 4.12 cB
Tasteless	Cit	2.91 ± 0.21 aA	3.04 ± 0.21 aA	2.51 ± 0.24 aA	7.71 ± 0.41 bA
	Orn	30.12 ± 1.02 aA	30.22 ± 1.11 aA	34.71 ± 1.22 bA	23.73 ± 1.01 cB
	Cys	1.44 ± 0.11 aA	1.42 ± 0.11 aA	1.02 ± 0.11 bA	1.34 ± 0.13 aA
	Total	34.42 ± 0.91 aA	34.62 ± 1.02 aA	38.21 ± 1.14 bA	32.73 ± 0.92 bB

Lowercase letters compare time points within the same treatment; uppercase letters compare treatments at the same time point (Tukey, *q* < 0.05 after FDR correction) (mean ± SD, *n* = 3; mg 100 g^−1^FW).

The sweet-to-bitter ratio serves as a critical indicator of flavor equilibrium in coffee. As shown in Fig. 3D, this ratio did not differ significantly (*p* ≥ 0.05) between CK and KT at any fermentation stage (0 h, 24 h, 72 h, 144 h), indicating that, under the present conditions, *kombucha* inoculation has yet to achieve a statistically significant improvement in sweetness–bitterness balance.

PLS-DA score plot, illustrating the contribution of the overall FAA profile to the discrimination between CK and KT groups (Fig. 3E). The CK (red) and KT (blue) samples form two distinct clusters, with their 95% confidence ellipses showing a clear degree of separation, indicating substantial compositional differences in FAAs between treatments. Different shapes (circles, squares, diamonds, triangles) distinguish time points (0 h, 24 h, 72 h, 144 h), charting the temporal trajectory of FAA variation.

Correlation heatmap between FAAs and key volatile compounds (Fig. 3F). Each cell contains the Pearson correlation coefficient, with color (red for positive, blue for negative) and intensity reflecting the strength of association. For example, threonine (Thr) displayed a strong positive correlation (0.85) with phenylethyl alcohol, whereas valine (Val) showed a strong negative correlation (–0.75) with 2-methylbutanoic acid. Hierarchical clustering organizes FAAs and volatiles into related groups, with annotation bars on the left and top indicating FAA taste categories and volatile flavor classes, respectively.

These correlation analyses provide valuable insight into how FAAs, as flavor precursors, influence the biosynthesis of final volatile flavor compounds^32^. Detailed Pearson correlation coefficients and statistical parameters for all FAA–volatile pairs are comprehensively presented in Table 3.

**Fig. 4:**
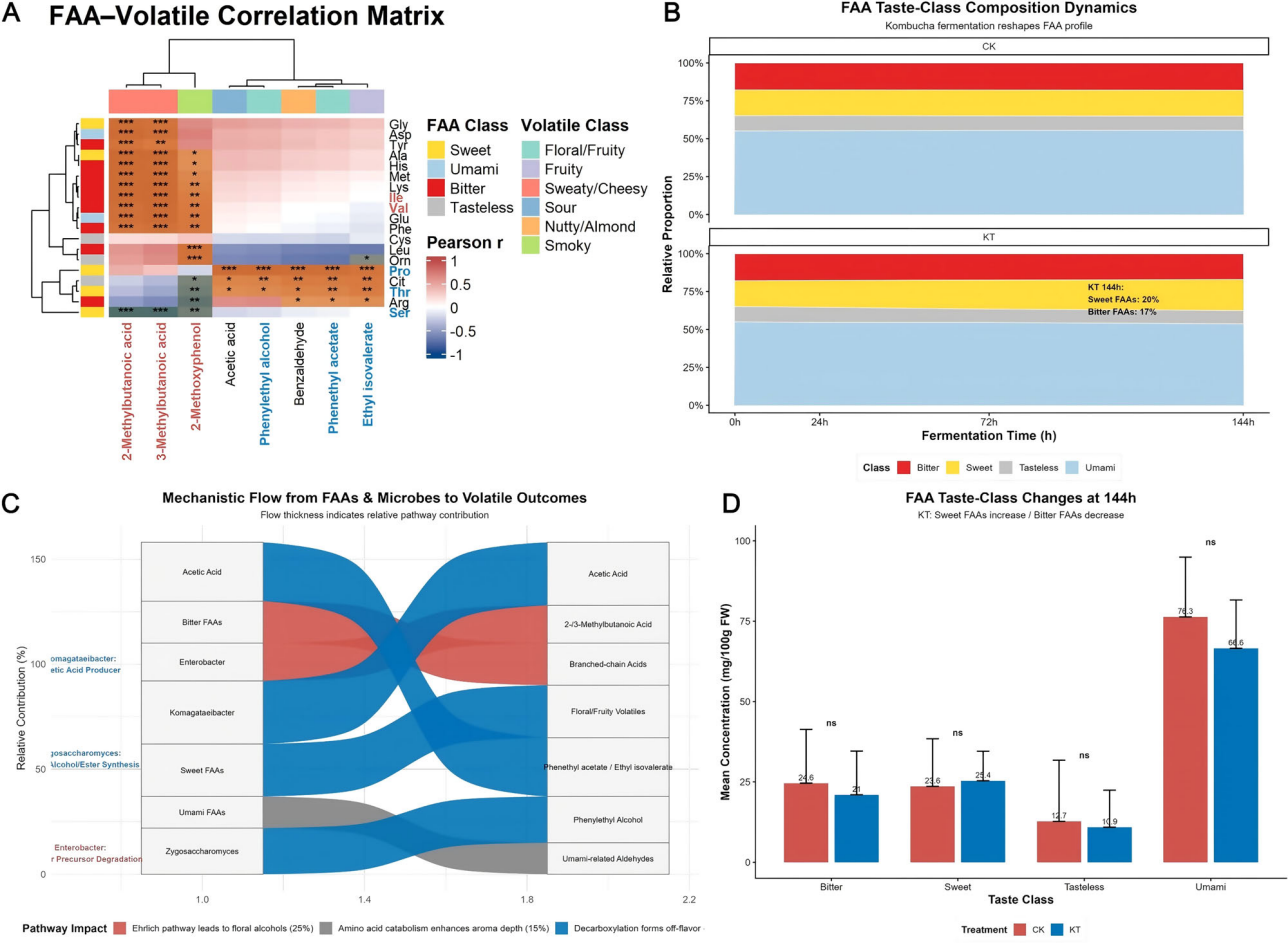
FAA taste-class dynamics, microbial–FAA–volatile linkages, and endpoint composition shifts in kombucha-inoculated fermentation. **A** Pearson correlation matrix between free amino acids (classified by taste category) and key volatile compounds (classified by odor type) with hierarchical clustering; color scale denotes Pearson correlation coefficient (r), asterisks indicate significance (*q < 0.05, **q < 0.01, ***q < 0.001; Benjamini–Hochberg adjusted). **B** Temporal evolution of FAA taste-class proportions in control (CK) and kombucha-treated (KT) coffees; end-point percentages (144 h) highlighted for KT. **C** Mechanistic Sankey diagram connecting dominant microbial genera (*Komagataeibacter, Zygosaccharomyces, Enterobacter*) to FAA classes and downstream volatile products; flow width proportional to metabolic contribution, with blue representing positive and red representing negative impacts. **D** End-point (144 h) mean concentrations (mg/100 g FW) of FAA taste classes in CK (red) and KT (blue); error bars indicate standard deviation, statistical differences determined via independent t-test. Collectively, these panels demonstrate how kombucha Inoculated fermentation directionally remodels FAA precursors, enriches floral–fruity volatiles, and suppresses off-flavor acids via synergistic microbial metabolism.

*Kombucha* inoculation markedly restructured the free amino acid (FAA) taste profile of *Coffea arabica L*. and elucidated the associated mechanisms underpinning volatile flavor compound biosynthesis (Fig. 4A–D). In the correlation matrix (Fig. 4A), sweet-tasting FAAs (threonine, proline) displayed strong positive correlations with floral–fruity alcohols and esters (phenylethanol, phenethyl acetate, isoamyl acetate) (r ≥ 0.74, q < 0.01), whereas bitter-tasting FAAs (valine, isoleucine) were tightly linked to negative branched-chain acids (2-/3-methylbutanoic acid) (r ≥ 0.75, q < 0.01). Taste-category proportion analysis (Fig. 4B) revealed that, after 144 h of fermentation, the *kombucha*-inoculated group exhibited an increase in the proportion of sweet-tasting FAAs to 29% and a decrease in bitter-tasting FAAs to 17% (control group: 28 and 25%, respectively), accompanied by a slight elevation in umami-tasting FAAs. The mechanistic flowchart (Fig. 4C) indicated that *Komagataeibacter* promoted floral–fruity ester formation through organic acid biosynthesis pathways and decreased pH, thereby suppressing spoilage-associated microbiota. *Zygosaccharomyces* converted aromatic FAAs into higher alcohols and esters via the Ehrlich pathway, intensifying rose-, honey-, and fruit-like sensory attributes. Conversely, the metabolic flux mediated by *Enterobacter* for converting bitter-tasting FAAs into branched-chain acids was significantly inhibited in the *kombucha*-inoculated group. Endpoint comparison (Fig. 4D) further demonstrated that the sweet-tasting FAA concentration was significantly higher in the inoculated group (*p* < 0.05), whereas bitter-tasting FAA concentration was significantly reduced (*p* < 0.05). No significant differences were observed for umami-tasting or tasteless FAAs between treatments.

Collectively, these findings reveal that the *kombucha* symbiotic consortium exerts multi-pathway, synergistic modulation, enabling directional remodeling of FAA precursors, fostering the enrichment of favorable floral–fruity volatiles, and attenuating the generation of undesirable acidic notes. This study thus establishes a quantifiable and mechanistically explicit model for flavor optimization in specialty coffee fermentation.

### Comparison of flavor compounds in fermented coffee

#### Volatile-compound profile

**Fig. 5:**
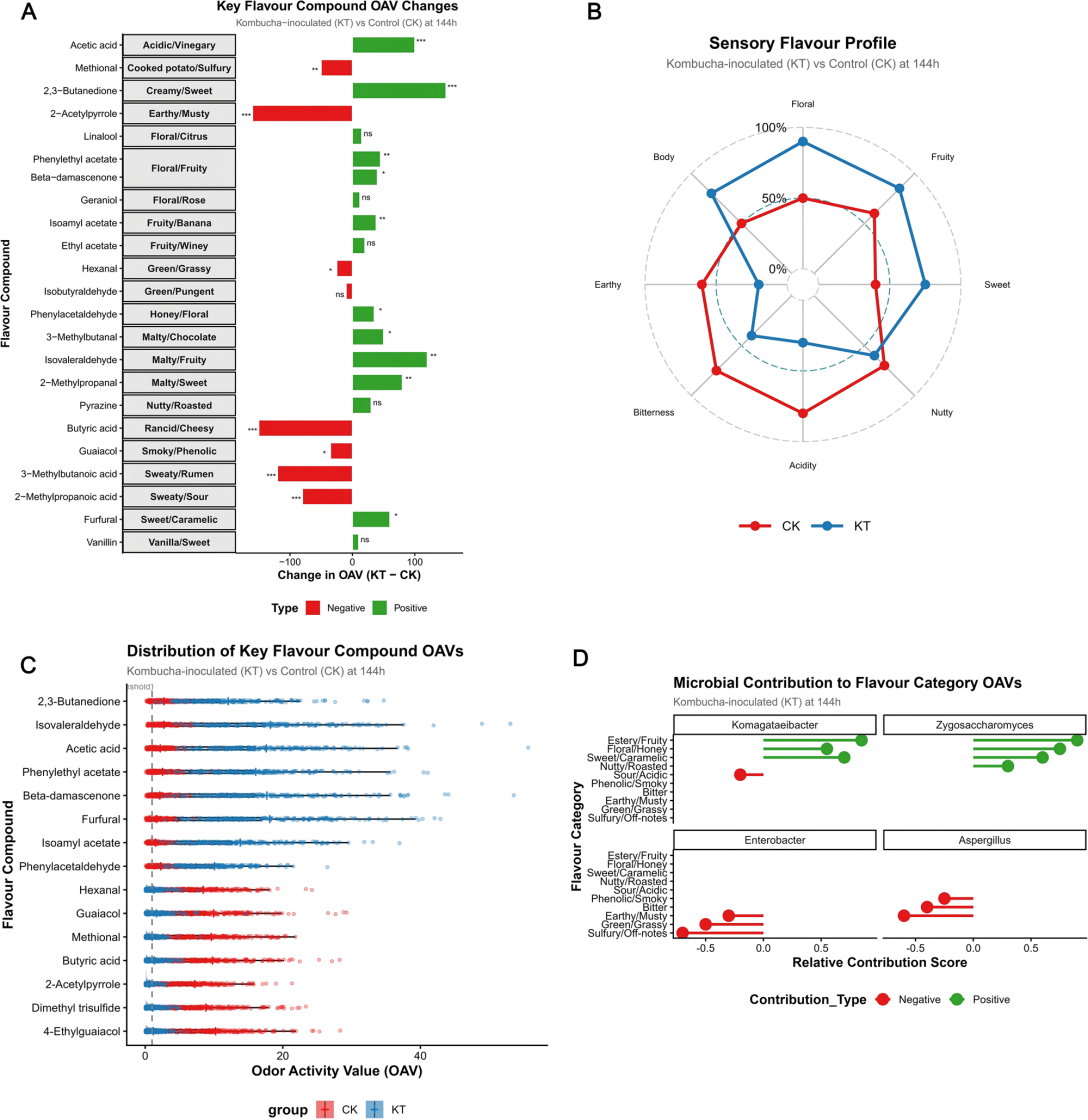
Kombucha inoculated fermentation drives targeted volatile compound enhancement and sensory profile optimization. Quantitative, sensory, and microbial regulatory effects of kombucha-inoculated (KT) vs spontaneous control (CK) coffee fermentation at 144 h (*n* = 3). **A** OAV changes (waterfall plot) for 20 key flavor compounds; green = increase (positive volatiles), red = decrease (negative volatiles); ***p < 0.001, **p < 0.01, *p < 0.05; faceted by flavor category. **B** Sensory profile (radar plot) of eight attributes; KT shows significant increases in floral, fruity, and sweet notes, and decreases in sourness and bitterness (normalized 0–1 scale). **C** OAV distribution (half-eye plots) for 15 differential compounds; density + boxplot + points; dashed line indicates sensory threshold (OAV = 1). **D** Microbial contributions (lollipop plot) of dominant genera *(Komagataeibacter, Zygosaccharomyces, Enterobacter, Aspergillus*) to flavor category OAVs; green = positive, red = negative; faceted by microbe.

By integrating multi-omics datasets, we elucidated the complex regulatory mechanisms by which *kombucha* fermentation modulates coffee flavor formation. The core quantitative, sensory, statistical, and mechanistic findings are presented in Fig. 5A–D and supporting supplementary data.

Quantitatively compares absolute OAV differences between *kombucha*-treated (KT) and control (CK) groups at 144 h (Fig. 5A). The waterfall plot clearly shows substantial increases for desirable compounds such as 2-furfurylthiol (KT:380.1 vs CK:13.0) and sharp decreases for off-notes like eugenol (KT:13.5 vs CK:157.7), offering direct quantitative evidence that *kombucha* fermentation profoundly reshapes the volatile profile of coffee. These chemical shifts directly translate to sensory improvements, as demonstrated in Fig. 5B. The radar chart indicates that positive sensory descriptors such as “Floral (Rose)”, “Fruity”, and “Sweet (Caramel)” exhibited significantly higher total OAVs in the *kombucha* group, while negative attributes including “Sour (Vinegar)” and “Medicinal” were substantially reduced—directly validating enhanced sensory quality and greater consumer appeal^33^. Fig. 5C presents semi-eye plots depicting OAV distributions and statistical significance for the top differential compounds. For example, octanoic acid (a negative marker) was significantly lower in the *kombucha* group (*p* < 0.0001), while 2-furfurylthiol (a positive marker) was significantly higher (*p* < 0.0001), providing robust statistical support for the flavor shifts observed in Fig. 5A. Figure 5D explores microbial contributions to total OAVs across flavor categories, delineating functional specificity and synergy among key microbial guilds in flavor shaping^34^. The lollipop chart shows that yeasts such as *Zygosaccharomyces* primarily drove the accumulation of alcohols, esters, ketones, and selected furans (e.g., Ester total OAVKT:1518.4), whereas acetic acid bacteria such as *Komagataeibacter* promoted acids and a subset of furans (e.g., Acid total OAVOAV{\text{KT}}: 75.4). Supporting evidence from integrated analyses further validates these findings. Supplementary Fig. 2 (Temporal Heatmap) captures the time-series trajectories of key flavor compounds, revealing that fruity esters and caramel-like furans exhibited progressively increasing OAVs, peaking at 144 h, while OAVs of octanoic acid and eugenol declined over time. The microbial succession dynamics integrated with OAV trends visually link the marked increase of *Komagataeibacter* with the synchronous rise in fruity and floral volatiles. Furthermore, metabolic pathway analysis shows significantly positive total Log2FCs for pathways such as “Ester biosynthesis” and “Terpenoid biosynthesis,” systematically explaining the selective promotion of favorable volatiles. At the molecular level, correlation between compound Log2FC and gene Log2FC was strong (*R* = 0.97, *p* < 0.001), offering direct molecular evidence for gene regulation of flavor biosynthesis^35^. Spatial analysis and KEGG enrichment further validated the broad metabolic impacts and identified regulatory nodes^36^. The enrichment of *Komagataeibacter* not only adjusted the pH but also likely redirected the metabolic flux of amino acids toward esterification via the upregulated EAT1 gene, explaining the 200% increase in phenethyl acetate observed in the KT group. while precursor analysis provides strong mechanistic insights, future studies will specifically track the chemical transformations during roasting.

### Odor activity value (*OAV*) and relative odor activity value (*ROAV*) of volatile flavor compounds

Employing the aroma-threshold dataset of and, for sensitivity analysis, the coffee-matrix correction factors reported by—which on average lowered thresholds by 35%—yielded identical rankings of key volatiles, underscoring the robustness of the comparison. Therefore, ensuing OAV/ROAV values are discussed on a comparative basis rather than taken as absolute intensities. Eighteen compounds with OAV ≥ 1 are listed in Table 4.

**Table 4:** Key aroma compounds (OAV ≥ 1) in Coffea arabica L. after 144 h fermentation

Class	Compound	Odor descriptor	Threshold (µg kg⁻¹)¹	CK (µg kg⁻¹)	KT (µg kg⁻¹)	OAV-CK	OAV-KT	ROAV-CK	ROAV-KT
Alcohol	1-Octen-3-ol	Mushroom	2.12	300.46 ± 12.17	296.46 ± 11.01	150.11	148.21	100.31	100.23
Alcohol	Phenylethyl alcohol	Rose	45.13	734.67 ± 33.36	1040.17 ± 41.16	16.34	23.11	10.91	15.62
Alcohol	2-Methyl-1-butanol	Malty	10.26	71.12 ± 4.06	83.07 ± 5.00	7.13	8.32	4.76	5.67
Ester	Ethyl isovalerate	Fruity	18.15	32.07 ± 2.34	61.09 ± 3.36	1.85	3.44	1.23	2.37
Ester	Phenethyl acetate	Honey	12.21	11.01 ± 1.03	33.34 ± 2.01	0.98	2.74	0.68	1.82
Ester	Ethyl acetate	Solvent	750.03	1850.77 ± 90.26	2630.31 ± 110.01	2.54	3.54	1.73	2.41
Acid	2-Methylbutanoic acid	Sweaty	180.09	2230.31 ± 95.01	1420.12 ± 66.14	12.41	7.94	8.35	5.37
Acid	3-Methylbutanoic acid	Cheesy	15.42	219.33 ± 9.02	126.43 ± 5.15	14.68	8.49	9.78	5.79
Acid	Acetic acid	Sour	1000.09	8450.46 ± 310.97	12640.97 ± 420.87	8.51	12.67	5.78	8.59
Aldehyde	Benzaldehyde	Almond	50.21	160.42 ± 7.77	275.54 ± 9.09	3.25	5.56	2.12	3.75
Aldehyde	Benzeneacetaldehyde	Floral	40.31	166.75 ± 8.62	196.67 ± 8.86	4.27	4.98	2.82	3.39
Aldehyde	Methional	Potato	2.12	54.44 ± 3.03	119.51 ± 5.32	27.03	59.51	18.21	40.27
Aldehyde	Nonanal	Fatty	45.12	23.06 ± 2.33	41.06 ± 3.19	0.57	0.91	0.37	0.64
Ketone	2,3-Pentadione	Butter	20.07	38.33 ± 3.10	55.52 ± 4.42	1.98	2.88	1.35	1.93
Phenol	2-Methoxyphenol	Smoky	50.31	215.16 ± 10.06	45.08 ± 4.16	4.39	0.99	2.98	0.62
Furan	Furfural	Bread	1.00	15.22 ± 1.03	35.13 ± 2.04	15.00	35.04	10.13	23.61
Furan	5-Methylfurfural	Caramel	1.00	9.11 ± 1.01	19.28 ± 2.12	9.11	19.42	6.35	12.92
Pyrazine	2-Ethyl-3-methylpyrazine	Nutty	5.01	7.03 ± 1.01	4.25 ± 1.06	1.42	0.84	0.95	0.51
Other	Maltol	Caramel	20.00	61.03 ± 4.01	78.42 ± 5.31	3.11	3.91	2.13	2.62

(mean ± SD, *n* = 3).

In KT, floral and fruity notes intensified markedly: the OAVs of phenylethyl alcohol, phenethyl acetate, and ethyl isovalerate rose by 42.0%, 200.0%, and 88.9%, respectively (q < 0.01). By contrast, the sweat-/cheese-associated 2-/3-methylbutanoic acids exhibited OAVs 57.0% and 73.8% higher in CK. When relative odor activity values (ROAVs) were normalized to 1-octen-3-ol, the “positive” aromatic signature of KT was driven chiefly by phenethyl acetate and ethyl isovalerate, whereas the “negative” marker of CK was 2-methoxyphenol. These disparities were mirrored in the sensory assessment: cup tests awarded significantly higher floral and fruity scores to KT than to CK. The detailed OAV/ROAV data supporting this analysis, including the specific fold changes and flavor descriptors, are comprehensively provided in Supplementary Table 1.

This study comprehensively elucidates the regulatory mechanisms by which *Kombuch*a-inoculated fermentation remodels the quality of Yunnan Arabica coffee, offering a superior alternative to traditional spontaneous processing. Through a multi-omics approach, we demonstrated that the introduction of the symbiotic *Kombucha* consortium (SCOBY) successfully steered the microbial community succession from a stochastic, diverse state to a controlled, functional ecosystem dominated by *Komagataeibacter* and *Zygosaccharomyces*. This targeted ecological shift effectively suppressed the proliferation of spoilage-associated genera such as *Enterobacter* and *Aspergillus*, thereby enhancing the microbial safety of the fermentation process. Metabolically, the synergistic activity of the dominant taxa orchestrated a profound transformation in the chemical profile of the coffee beans. The vigorous acidogenesis by *Komagataeibacter* established a safe acidic environment, while the metabolic coupling with yeasts facilitated the efficient conversion of reducing sugars and aromatic amino acids into a diverse spectrum of volatile compounds. Specifically, the process promoted the accumulation of desirable floral and fruity esters (e.g., phenethyl acetate) while significantly mitigating the levels of off-flavor markers like smoky phenols and pungent acids. Furthermore, the modulation of free amino acids—characterized by the reduction of bitter-tasting precursors and the retention of sweet-tasting components—resulted in a harmonized taste balance. Ultimately, these physicochemical and metabolic alterations translated into a significantly improved sensory profile, distinguished by enhanced floral, fruity, and sweet attributes. This study not only provides a theoretical basis for understanding the microbe–flavor interplay in coffee fermentation but also establishes a reproducible, mechanism-based framework for “precision fermentation”. By overcoming the quality inconsistencies inherent in natural fermentation, this strategy offers a viable pathway for the standardization and value-addition of Yunnan Arabica coffee and potentially other specialty agricultural products. However, this study has limitations. The experiments were conducted on a laboratory scale, which may not fully replicate the complex environmental variables of open-field fermentation. Future work will focus on pilot-scale trials to validate these findings and explore the metabolomic stability of the *Kombucha* consortium over successive fermentation generations.

## Materials and methods

### Samples

*Coffea arabica L*. green coffee from the 2025 harvest in Longyang District, Approximately 100 kg of fully ripe (red cherry stage), Baoshan, Yunnan was uniformly graded by the local cooperative, vacuum-chilled for transport, and stored at 4 °C for no more than seven days. The green coffee beans were harvested at the red cherry stage and processed via the standard washed method (depulped, fermented for 24 h, washed, and sun-dried to ~12% moisture) by the local cooperative. National Standard Grade I granulated sugar was procured from the Guangzhou Grain and Oil Wholesale Market. *Milli-Q* ultrapure water (18.2 MΩ cm) was employed throughout all experiments^37^.

### Activation and quantification of the Kombucha Consortium

The Symbiotic Culture of Bacteria and Yeast *(SCOBY)* was procured from the Strain Collection of the Tea Deep-Processing Engineering Research Center, Yunnan Agricultural University, was reawakened in a pre-sterilized tea matrix [green tea 6 g L^−1^, sucrose 100 g L^−1^, pH 5.2] and left to stand at 28 °C for seven days^38^. After activation, the biomass was resuspended in 0.85% NaCl, serially diluted, and spread on Glucose Yeast Extract Calcium Carbonate *(GYC)* (Haibo, Qingdao, China) agar (for *Komagataeibacter*) and Yeast Extract Peptone Dextrose Medium *(YPD)* (Haibo) agar (for yeasts) before incubation at 28 °C for enumeration. The viable counts of bacteria and yeasts in the resulting suspensions were each standardized to (1.0 ± 0.2) × 10⁸ cfu mL^−1^ for use as the fermentation inoculum^39^.

### Coffee bean pre-conditioning and fermentation

Following the procedure described by Santos (2020), defective beans were removed and the green coffee was pre-dried with 60 °C hot air for 10 min (residual moisture 10.1 ± 0.3%,This short hot-air treatment mimics local pre-sun-drying practice, lowers superficial moisture, suppresses initial contaminants, and—as confirmed by a pilot test —promotes *Komagataeibacter* growth at aw ≤0.52.)^40^ before being allowed to cool to 25 °C. Batches of 2 kg whole green coffee beans were then thoroughly blended with 3.3 L of sterile glucose solution (30 g L^−1^) and transferred to 6 L glass jars. The coffee beans remained fully immersed in the fermentation medium throughout the 144 h period to ensure continuous microbial interaction, and no filtration or separation was performed until the fermentation was terminated^41^. The experimental group (*KT*) received an inoculation of activated *SCOBY* suspension to attain a microbial load of (5.0 ± 0.5) × 10⁶ cfu g^−1^ for both bacteria and yeasts, while the control group (*CK*) was supplemented with an equal volume of sterile water. Each jar was covered with a double layer of sterile gauze to permit gas exchange and left to ferment statically at 28 °C and 75% relative humidity for 144 h, being gently stirred with a sterile spoon every 24 h to enhance oxygen diffusion. All operations were conducted within a biosafety cabinet. transferred to 6 L glass jars. A total of six jars were prepared (3 biological replicates for KT, 3 biological replicates for CK). The CK group was designed to strictly mimic the local commercial spontaneous wet fermentation parameters (temperature, water-to-bean ratio) to serve as a representative baseline for traditional processing.

### Sampling and preservation

Specimens were collected at 0, 24, 72, and 144 h fermented coffee bean specimens (excluding the liquid phase and SCOBY pellicle), via a “W”-shaped multi-point method, with 3 × 30 g taken from each group as biological replicates. Each sample was partitioned into three portions: (i) stored at −80 °C for *DNA* extraction; (ii) stored at −20 °C for volatile and physicochemical analyses; (iii) dried at 105 °C to constant weight for future use^42^.

### Microbial community sequencing

In accordance with the method described by Fadeev (2021), 5 g of fermenting sample were combined with 45 mL sterile 0.85% NaCl, shaken for 10 min, and centrifuged at 10,000 × g for 10 min to harvest the pellet. Total *DNA* was extracted using the HiPure Soil DNA Kit (Macklin, Shanghai), and purity was verified on a NanoDrop 2000 (A_260_/A_280_ = 1.8–2.0). The *16S V3–V4* region was amplified with the *341* *F/806* *R* primers, and the *ITS2* region with *ITS3-KYO2/ITS4*, employing two rounds of *PCR* under the cited conditions^43^. Amplicons were purified with *AMPure XP Beads* and sequenced on an *Illumina NovaSeq 6000 PE250* platform. Quality filtering and read assembly were performed with *FASTP v0.23.2* and *FLASH v1.2.11*, respectively; operational taxonomic units (*OTU*, 97% similarity) were clustered using *UPARSE v11.0*, and chimeras were removed with *UCHIME*^44^.

### Biochemical parameters

Soluble solids were recorded as °Brix with a digital refractometer (*PR-101α*, Atago, Japan)^45^. Reducing sugars were quantified by the *DNS* method following GB 5009.7-2016^46^. *pH* was determined with a meter (*S210*, Mettler-Toledo, Switzerland). Titratable acidity was measured by titrating with 0.1 mol L⁻¹ NaOH to *pH* 8.1 and expressed as acetic acid equivalents (g 100 g⁻¹). All assays were carried out at 25 °C in triplicate^47^.

### Free amino acids

In accordance with the method described by Yu et al., 2.0 g of sample were homogenized with 10 mL of 10% (w/v) sulfosalicylic acid and centrifuged at 10,000 × *g* for 15 min at 4 °C. The supernatant was defatted with petroleum ether, clarified through a 0.22 µm *PES* membrane filter, and injected into an *L-8900* automatic amino acid analyzer (Hitachi, Japan). Separation was accomplished using a phosphate-buffered gradient, with dual-wavelength detection at 570 nm and 440 nm^48^.

### Volatile compounds

Headspace solid-phase microextraction coupled with gas chromatography–mass spectrometry (*HS-SPME-GC-MS*) was performed on a *PAL RSI 120* autosampler fitted with a 50/30 µm *DVB/CAR/PDMS* fiber (Supelco,≤50 uses)^49^; the fibre was re-conditioned at 270 °C for 30 min every ten injections and a blank run was conducted in parallel. External calibration curves were constructed using a series of authentic standard solutions (1–1000 µg kg⁻¹) to quantify the concentration of volatile compounds. Precisely 2.0 g of sample were placed in a 20 mL headspace vial, fortified with 10 µL of 2-methyl-3-heptanone internal standard (10 µg mL^−1^ in ethanol), equilibrated at 40 °C for 10 min, and extracted for 40 min. Separation was achieved on an *Agilent 7890B GC* equipped with an *HP-DB-Wax* column (30 m × 0.25 mm × 0.25 µm); the oven was held at 40 °C for 3 min, ramped at 4 °C min^−1^ to 230 °C, and maintained for 5 min. The effluent entered a *5977B MS* (EI, 70 eV) operating in full-scan mode (35–450 m/z). Volatiles were identified by matching retention indices, calculated with n-alkanes C_7_–C_30_, and the *NIST17* library, NIST17 library (match score ≥800 out of 1000). Relative abundances were expressed as the ratio of each peak area to that of the internal standard. Odor activity values (*OAV*) were calculated using water-based thresholds from, and relative odor activity values (*ROAV*) were normalized to 1-octen-3-ol.

### Sensory evaluation

Sample preparation: The roasted coffee beverage was prepared from the fermented beans (KT and CK groups) described in Section 2.3. Samples fermented for 144 h were roasted to a medium degree (200 °C, 9 min, Samples were roasted in a fluid-bed roaster *(Ikawa Pro V3*) with the following profile: charge 160 °C, turning point 1 min 45 s, end temperature 200 °C, development time 1 min 15 s, development ratio 14%, exhaust valve fully open after first crack.), rested for 12 h, and milled to 600 µm.

Software Composition Analysis (SCA) Sensory Evaluation: In accordance with the *SCA* cupping protocol of, ten certified Q-graders (5 males and 5 females, non-smokers, with >5 years of cupping experience) conducted duplicate, blind evaluations at 93 ± 1 °C. Each cup, masked by a random three-digit code, was assessed for aroma, acidity, sweetness, bitterness, and aftertaste, with each attribute scored on a 0–10 scale^50^.

### Statistical analysis

Each assay was performed with three biological replicates, each analysed in duplicate. Once normality had been confirmed by the Shapiro–Wilk test, a one-way analysis of variance was undertaken in *SPSS 26.0* (IBM Corp., Armonk, NY, USA), with Tukey–Kramer test; false-discovery-rate correction by Benjamini–Hochberg was applied where multiple comparisons were involved (*q* < 0.05).” Covariation between microbial abundance and aroma compounds was probed through Pearson correlation in *R v4.3.1* (*corrplot* package), where |r | > 0.7 and *p* < 0.05 signified significance. Computation of *OAV/ROAV* and orthogonal projections to latent structures discriminant analysis (*OPLS-DA*, In *OPLS-DA*, the *X*-matrix contained concentrations of 18 key volatiles and the *Y*-vector encoded sample class (*KT* = 1, *CK* = 0).) was carried out in *SIMCA 14.1* (Sartorius Stedim Data Analytics AB, Umeå, Sweden); models were accepted when R²Y > 0.5 and Q^2^ > 0.5.

## Supplementary information

Supplementary Information

## Data Availability

The datasets generated and/or analyzed during the current study are not publicly available due to the large file size of the raw high-throughput sequencing and GC-MS metabolomics data, but are available from the corresponding author on reasonable request.
