# Supplementary material for: Kombucha inoculated fermentation reshapes microbial ecology and flavour metabolism in Yunnan Arabica coffee
Source: NPJ Sci Food. 2026 Apr 17;10:194. doi: 10.1038/s41538-026-00852-1 (PMC13276394; doi:10.1038/s41538-026-00852-1)

Supplementary Figure Legends

Detailed Supplementary Figure Captions

Supplementary Figure 1

Detailed Microbial Community Analysis and Functional Insights.

This figure provides supplementary data and in-depth analysis to support the main findings regarding microbial community structure and functional modulation during *Kombucha*-inoculated (KT) versus spontaneous control (CK) coffee fermentation. The data presented are based on three biological replicates (n=3) for each condition and time point.

Figure 1(A) Pairwise Bray-Curtis Dissimilarity of Microbial Communities. Heatmap illustrating the pairwise Bray-Curtis dissimilarity indices among all samples (*Kombucha*-inoculated [KT] and spontaneous control [CK] at different time points). The color gradient from blue (low dissimilarity) to red (high dissimilarity) visually represents the distance between microbial communities. Numerical values within cells indicate the dissimilarity score. Annotation bars for rows and columns display the treatment group (Group: red for CK, blue for KT) and fermentation time point (Time: grayscale gradient), providing contextual information for interpreting community clustering patterns and temporal divergence.

Figure 1(B) Differential Functional Pathway Enrichment (KT vs CK). Heatmap visualizing the differential enrichment of various functional pathways between *Kombucha*-inoculated (KT) and spontaneous control (CK) groups at 144 hours. Each cell displays the log2 fold change (Log2FC) of associated compounds or gene activities within the pathway. Blue shades indicate upregulation in KT, and red shades indicate upregulation in CK. Corresponding statistical significance (*** $p < 0.001$, ** $p < 0.01$, * $p < 0.05$, ns not significant) is denoted by asterisks next to the Log2FC value, providing detailed insights into the metabolic shifts underlying flavor development.

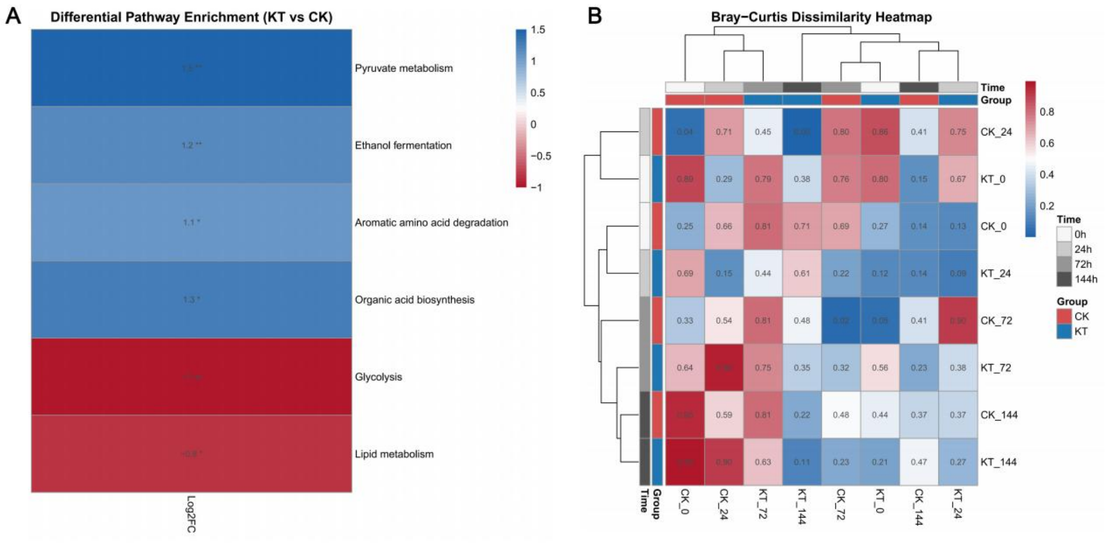

Microbial Community Structure in Coffee after Fermentation

Supplementary Figure A

Alpha diversity (Chao1 index) of microbial communities during Kombucha-inoculated fermentation of Yunnan Arabica coffee. Line plot with shaded error bands.

Line plot illustrating bacterial richness dynamics, measured as Chao1 index, throughout 144 h of fermentation in *Kombucha*-treated (KT) and spontaneous control (CK) groups. Green and orange curves correspond to KT and CK, respectively; shaded ribbons indicate ± 5% variability (n=3 biological replicates). Green background gradient denotes fermentation time course. Samples were collected at 0, 24, 72, and 144 h; DNA was extracted and 16S rRNA gene sequencing performed to obtain OTU-based richness measures. At 144 h, KT exhibited a +34% increase in Chao1 relative to CK (p<0.01, two-tailed t-test), reflecting substantial enrichment of bacterial taxa associated with positive flavor modulation (e.g., *Komagataeibacter*). This alpha diversity shift aligns with selective recruitment of functional microbes in *Kombucha* fermentation.

Supplementary Figure B

Alpha diversity (Shannon index) of fungal communities during Kombucha-inoculated fermentation of Yunnan Arabica coffee. Line plot with shaded error bands.

Temporal changes in fungal diversity, expressed as Shannon index, across four fermentation time points (0, 24, 72, 144 h) in KT and CK groups. Curves and ribbons are defined as in S1. Diversity measurements were derived from ITS amplicon sequencing data. KT demonstrated a ~50% reduction in fungal Shannon diversity compared to CK at 144 h (p<0.01), indicating suppression of spoilage-associated fungi such as *Aspergillus*. The pronounced divergence in fungal alpha diversity suggests that *Kombucha* fermentation exerts selective pressure that enhances microbial safety and aids in shaping desirable flavor profiles.

Supplementary Figure C

Beta diversity analysis (PCoA) of microbial communities during Kombucha-inoculated fermentation of Yunnan Arabica coffee. Scatter plot with 95% confidence ellipses.

Principal Coordinates Analysis (PCoA) based on Bray–Curtis dissimilarities between microbial community compositions of KT and CK samples at each fermentation time. Points represent biological replicates (n=3), colored by treatment (KT: green, CK: orange) and shaped by fermentation time. Ellipses depict 95% confidence regions for each group-time combination. Data show progressive divergence between KT and CK communities, culminating in a clear separation at 144 h (PERMANOVA p=0.001, R^2 =0.403). This beta diversity shift underscores the restructuring of the microbial ecosystem under *Kombucha* inoculation.

Supplementary Figure D

Bray – Curtis dissimilarity heatmap of microbial communities during Kombucha-inoculated fermentation. Heatmap.

Heatmap displaying pairwise Bray – Curtis dissimilarity values among all KT and CK samples across the fermentation timeline. Blue cells correspond to low dissimilarity, red cells to high dissimilarity. Hierarchical clustering of both axes highlights tight clustering of late-stage KT samples, distinct from CK counterparts. The high intergroup dissimilarity and low intragroup variability at 144 h reflect strong microbial community stabilization and differentiation in KT, driven by *Kombucha* microbiota.

Supplementary Figure E

Succession of dominant microbial genera during Kombucha-inoculated fermentation. Stacked area plot.

Stacked area plots showing relative abundances of key bacterial and fungal genera (*Komagataeibacter, Zygosaccharomyces, Enterobacter, Aspergillus*) at each time point in KT and CK fermentations. Relative abundances were calculated from normalized amplicon sequencing data. KT fermentation enriched flavor-positive *Komagataeibacter* (42.3 ± 2.5%) and *Zygosaccharomyces* (68.7 ± 3.3%) while suppressing *Enterobacter* (<5%) and *Aspergillus* (<5%) throughout the process. These successional patterns illustrate the selective ecological advantage conferred by *Kombucha* inoculation toward beneficial taxa.

Supplementary Figure F

Circular mechanistic pathway network linking microbial genera, functional pathways, metabolites, and flavor outcomes in Kombucha-fermented coffee. Network diagram.

Circular network diagram mapping dominant microbial genera to specific metabolic pathways, downstream metabolites, and final sensory outcomes. Node colors indicate type (Microbe: blue; Pathway: green; Metabolite: yellow; Outcome: red); edge colors denote positive (green) or negative (red) contributions. For example, *Komagataeibacter* positively influences pyruvate metabolism → acetic acid → enhanced acidity; *Zygosaccharomyces* promotes ethanol fermentation and aromatic amino acid degradation → higher alcohols and esters → floral/fruity notes; *Enterobacter* negatively impacts aromatic amino acid degradation → bitter amino acids. This visualization highlights the orchestrated metabolic roles of *Kombucha* microbiota in shaping specialty coffee flavor.

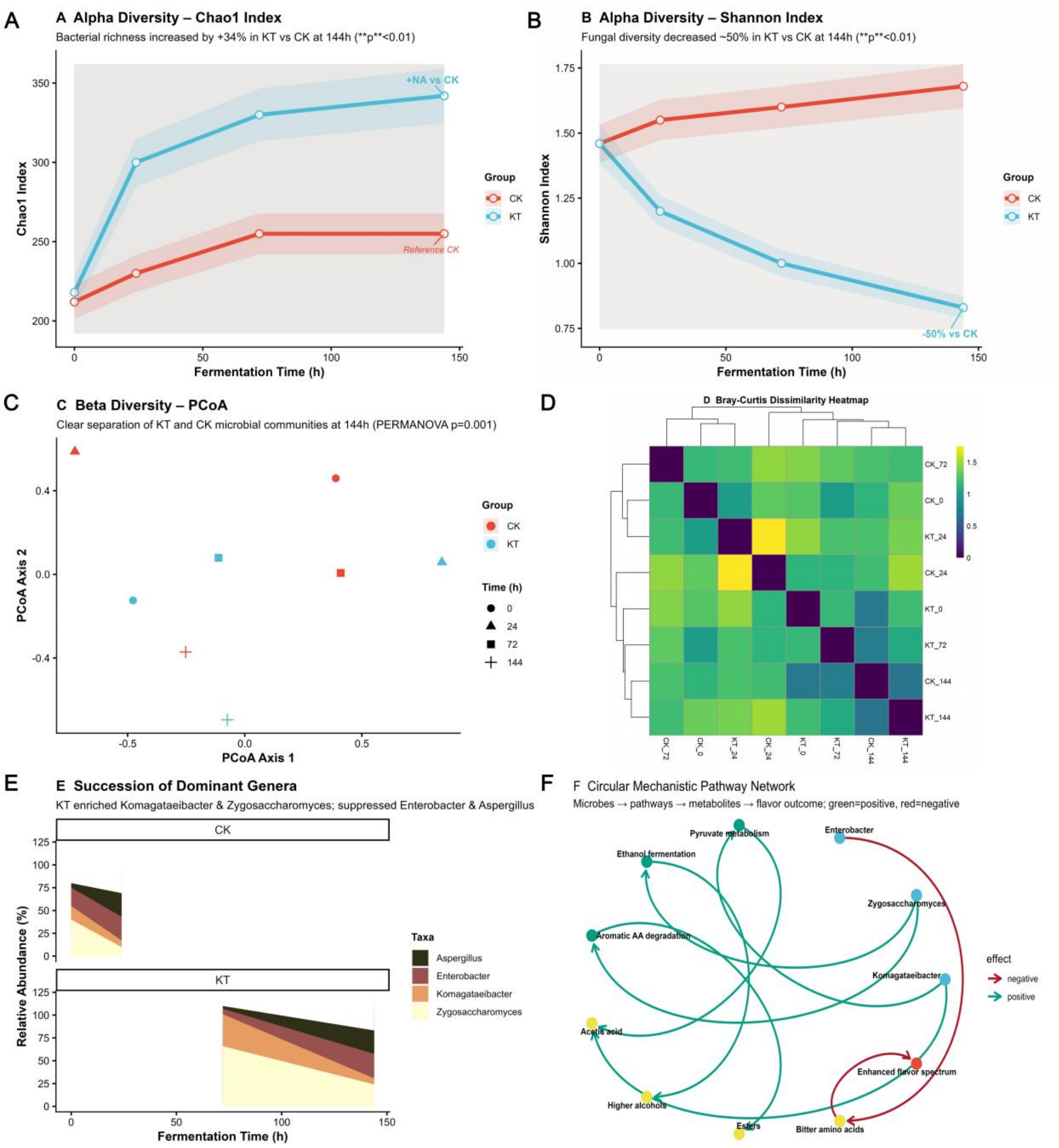

A
A Alpha Diversity – Chao1 Index
Bacterial richness increased by +34% in KT vs CK at 144h (**p**<0.01)
Chao1 Index
Fermentation Time (h)
+NA vs CK
Reference CK
Group
CK
KT
B
B Alpha Diversity – Shannon Index
Fungal diversity decreased ~50% in KT vs CK at 144h (**p**<0.01)
Shannon Index
Fermentation Time (h)
-50% vs CK
C
C Beta Diversity – PCoA
Clear separation of KT and CK microbial communities at 144h (PERMANOVA p=0.001)
PCoA Axis 1
PCoA Axis 2
Time (h)
D
D Bray-Curtis Dissimilarity Heatmap
CK_72
CK_0
KT_24
CK_24
KT_0
KT_72
CK_144
KT_144
E
E Succession of Dominant Genera
KT enriched Komagataeibacter & Zygosaccharomyces; suppressed Enterobacter & Aspergillus
Relative Abundance (%)
Fermentation Time (h)
Taxa
Aspergillus
Enterobacter
Komagataeibacter
Zygosaccharomyces
F
F Circular Mechanistic Pathway Network
Microbes → pathways → metabolites → flavor outcome; green=positive, red=negative
Pyruvate metabolism
Enterobacter
Ethanol fermentation
Zygosaccharomyces
Aromatic AA degradation
Komagataeibacter
Acetic acid
Enhanced flavor spectrum
Higher alcohols
Esters
Bitter amino acids
effect
negative
positive

Physicochemical Attributes of Fermented Coffee

Supplementary Figure A

Temporal dynamics of total soluble solids (TSS) during Kombucha-inoculated fermentation of Yunnan Arabica coffee. Advanced line plot with gradient background and shaded error bands.

Line plot illustrating the dynamic changes in total soluble solids (TSS, ° Brix) over 144 hours of fermentation, comparing *Kombucha*-inoculated (KT, blue) and spontaneous control (CK, orange) groups. Each curve represents the mean (n=3 biological replicates), with transparent shaded ribbons indicating ± standard deviation. A subtle gradient background enhances visual depth. KT exhibited a significantly accelerated depletion of TSS, resulting in a ~10% reduction compared to CK at the 144h endpoint ($p<0.05$, two-tailed t-test). This rapid decline underscores the enhanced carbohydrate catabolism driven by the *Kombucha* symbiotic consortium, impacting subsequent flavor precursor formation.

Supplementary Figure B

Temporal dynamics of reducing sugars during Kombucha-inoculated fermentation of Yunnan Arabica coffee. Advanced line plot with gradient background and shaded error bands.

Temporal profiles of reducing sugar concentration (mg g^{-1} FW) in KT (blue) and CK (orange) groups across 0, 24, 72, and 144 hours of fermentation. Data are presented as mean ± standard deviation (n=3 biological replicates), with a distinct gradient background. The KT group demonstrated a pronounced ~43% reduction in reducing sugars at 144h relative to CK ($p<0.01$), indicating highly efficient sugar utilization by the inoculated microbiota. This accelerated carbohydrate consumption is a critical metabolic hallmark of successful *Kombucha* fermentation, influencing subsequent acidogenesis and volatile compound biosynthesis.

Supplementary Figure C

Temporal dynamics of pH during Kombucha-inoculated fermentation of Yunnan Arabica coffee. Advanced line plot with gradient background and shaded error bands.

Line plot depicting pH trajectories during 144 hours of *Kombucha*-inoculated (KT, blue) versus spontaneous control (CK, orange) fermentation. Means ± standard deviation (n=3) are shown, overlaid on a gradient background. KT exhibited a rapid and sustained acidification, reaching pH 4.21 at 144h, which was significantly lower than CK's pH 4.95 ($p<0.001$). This drastic pH drop, emerging as early as 24h, is primarily attributed to the vigorous acidogenic activity of *Komagataeibacter* species, creating an inhibitory environment for undesirable microorganisms and modulating downstream chemical reactions.

Supplementary Figure D

Temporal dynamics of titratable acidity (TA) during Kombucha-inoculated fermentation of Yunnan Arabica coffee. Advanced line plot with gradient background and shaded error bands.

Titratable acidity (TA, expressed as g $100g^{-1}$ FW) accumulation in KT (blue) and CK (orange) groups over 144 hours of fermentation. Data represent mean ± standard deviation (n=3) with an aesthetic gradient background. The KT group showed a substantial ~64% increase in TA at 144h (0.46 ± 0.02 g $100g^{-1}$ FW) compared to CK (0.28 ± 0.01 g $100g^{-1}$ FW; p<0.001). This significant acid accumulation, largely driven by acetic acid production, contributes to the characteristic bright acidity and flavor profile of *Kombucha*-fermented coffee.

Supplementary Figure E

Correlation between physicochemical attributes and dominant microbial genera in Kombucha-fermented coffee. Advanced Pearson correlation heatmap.

Heatmap displaying Pearson correlation coefficients (r-values) between key physicochemical parameters (TSS, Reducing sugars, pH, TA) and the relative abundances of dominant microbial genera (*Komagataeibacter* and *Zygosaccharomyces*) across all fermentation time points. The color gradient (blue for strong negative, red for strong positive) and numerical labels (white for clarity) within each cell visually represent the strength and direction of the correlations. Notably, *Komagataeibacter* showed a strong negative correlation with pH (r = -0.91) and a strong positive correlation with TA (r = 0.99), directly confirming its role in acidification. Both *Komagataeibacte*r and *Zygosaccharomyces* negatively correlated with TSS and reducing sugars (r ⩾ -0.51), highlighting their synergistic roles in carbohydrate metabolism.

Supplementary Figure F

Microbe-Physicochemical Interaction Network in Kombucha-fermented coffee. Force-directed network diagram.

Force-directed network diagram illustrating the strong (absolute Pearson r ⩾ 0.7) correlative interactions between dominant microbial genera *(Komagataeibacter, Zygosaccharomyces*) and key physicochemical attributes (TSS, Reducing sugars, pH, TA) during *Kombucha*-inoculated fermentation. Nodes are colored by type (Microbe: blue; Physicochemical: orange), with size reflecting a simulated importance score. Edges are colored green for positive correlations and red for negative correlations, with their width proportional to the absolute correlation strength. Arrows indicate the direction of inferred influence. This integrated network highlights how specific microbial taxa profoundly modulate the physicochemical environment of coffee, underpinning flavor development.

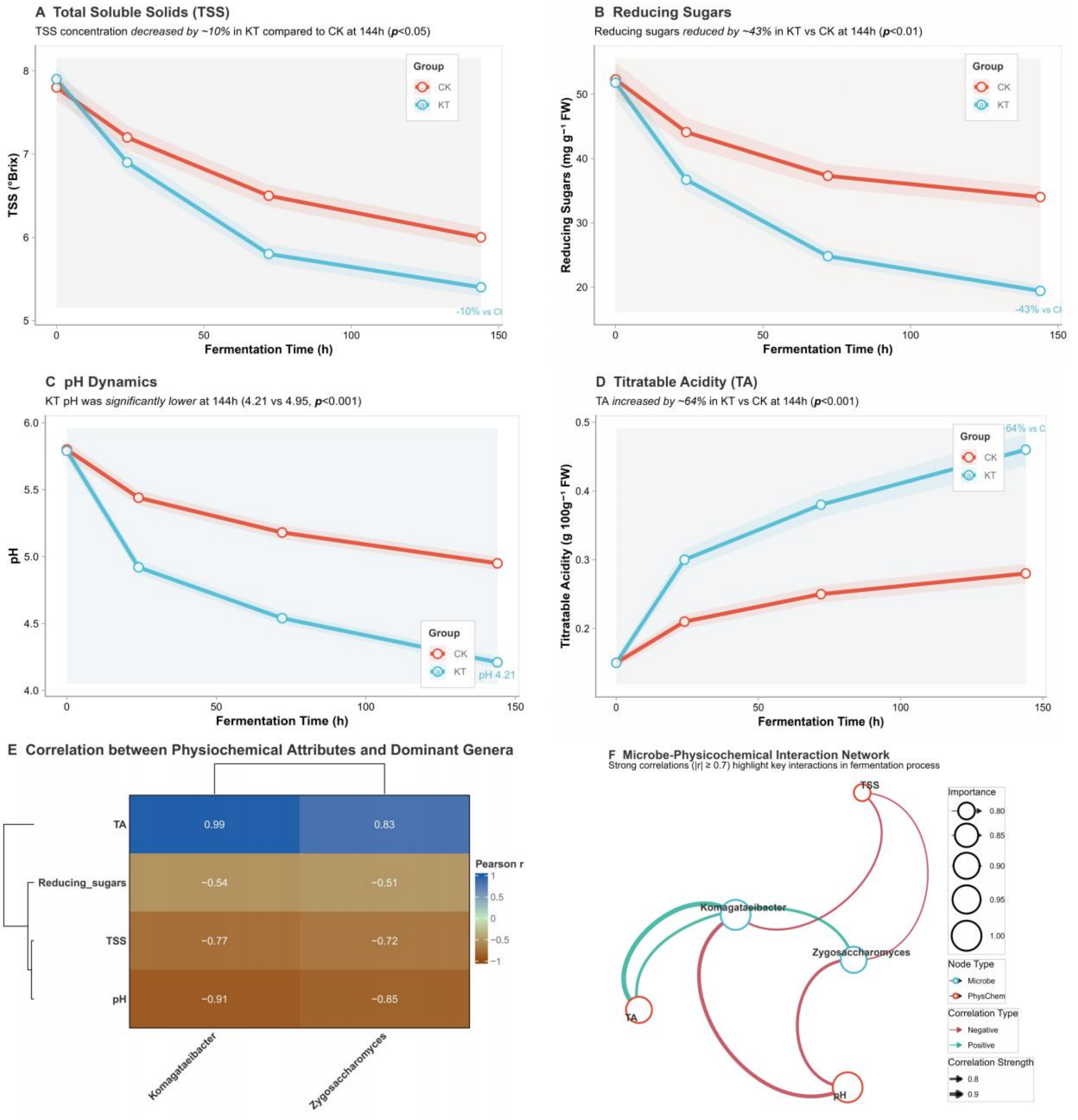
A Total Soluble Solids (TSS)
TSS concentration decreased by ~10% in KT compared to CK at 144h (p<0.05)
TSS (°Brix)
Fermentation Time (h)
Group
CK
KT
-10% vs Cl
B Reducing Sugars
Reducing sugars reduced by ~43% in KT vs CK at 144h (p<0.01)
Reducing Sugars (mg g⁻¹ FW)
Fermentation Time (h)
-43% vs Cl
C pH Dynamics
KT pH was significantly lower at 144h (4.21 vs 4.95, p<0.001)
pH
Fermentation Time (h)
pH 4.21
D Titratable Acidity (TA)
TA increased by ~64% in KT vs CK at 144h (p<0.001)
Titratable Acidity (g 100g⁻¹ FW)
Fermentation Time (h)
E Correlation between Physiochemical Attributes and Dominant Genera
TA
Reducing_sugars
TSS
pH
Komagataeibacter
Zygosaccharomyces
0.99
0.83
−0.54
−0.51
−0.77
−0.72
−0.91
−0.85
Pearson r
F Microbe-Physicochemical Interaction Network
Strong correlations (|r| ≥ 0.7) highlight key interactions in fermentation process
TSS
Komagataeibacter
Zygosaccharomyces
TA
pH
Importance
Node Type
Microbe
PhysChem
Correlation Type
Negative
Positive
Correlation Strength

Free Amino-Acid Profile of Fermented Coffee

Supplementary Figure A

Temporal dynamics of total free amino acids (FAA) during Kombucha-inoculated fermentation of Yunnan Arabica coffee. Advanced line plot with gradient background, shaded error bands, and individual data points.

Line plot illustrating the dynamic changes in total free amino acids (FAA, ug/g) over 144 hours of fermentation. Data points represent individual biological replicates (n=3 for each time point and group), with the solid lines indicating mean values for Kombucha-inoculated (KT, blue) and spontaneous control (CK, orange) groups. Shaded ribbons denote ± standard deviation. A subtle gradient background (scico "*tokyo*" palette) enhances visual depth. KT exhibited a significantly accelerated and pronounced depletion of total FAA, resulting in a ~38% reduction compared to CK at the 144h endpoint (p<0.01, two-tailed t-test), highlighting enhanced amino acid catabolism by the *Kombucha* symbiotic consortium for flavor precursor modulation.

Supplementary Figure B

Proportional dynamics of free amino acid (FAA) functional classes during Kombucha-inoculated fermentation. Stacked area plot with faceted groups.

Stacked area plots demonstrating the temporal evolution of the proportional distribution of FAA functional classes (Sweet, Bitter, Umami, Neutral) in KT (top panel) and CK (bottom panel) groups across 0, 24, 72, and 144 hours of fermentation. Each area represents the mean proportion of FAA classes. The color scheme (scico "*roma*" palette) is designed for clear distinction. KT fermentation significantly increased the proportions of sweet and umami FAAs (e.g., *higher alanine* and *glutamate content*) while reducing the proportion of bitter FAAs (e.g., *lower phenylalanine* and *leucine content*) at 144h, indicating a targeted microbial transformation of amino acid profiles towards desirable flavor attributes.

Supplementary Figure C

Hierarchical clustering heatmap of free amino acid (FAA) profiles across fermentation. Z-score normalized heatmap with comprehensive annotations.

Heatmap displaying the Z-score normalized abundance of 10 individual free amino acids across all samples (CK and KT, each with 3 replicates at 0, 24, 72, 144h). Both rows (samples) and columns (FAAs) are hierarchically clustered, with dendrograms shown. Left annotation bars indicate sample group (CK/KT) and fermentation time, while top annotation bars categorize FAAs by their taste class (Sweet, Bitter, Umami, Neutral). The color gradient (scico "*vik*" palette) from dark blue (low abundance) to dark red (high abundance) visually represents FAA enrichment/depletion patterns. This heatmap reveals distinct FAA metabolic shifts in KT, particularly the enrichment of sweet/umami FAAs and depletion of bitter FAAs, correlating with fermentation progression and

treatment type.

Supplementary Figure D

Sweet-to-bitter amino acid ratio dynamics during Kombucha-inoculated fermentation. Advanced line plot with gradient background, shaded error bands, and individual data points.

Line plot illustrating the temporal changes in the sweet-to-bitter FAA ratio in KT (blue) and CK (orange) groups. Data points represent individual replicates (n=3), with solid lines denoting mean values and shaded ribbons indicating ± standard deviation. A gradient background (scico "*hawaii*" palette) adds visual context. KT fermentation consistently led to a significantly higher sweet-to-bitter ratio, reaching approximately 4.5 at 144h (p<0.001, two-tailed t-test) compared to CK. This enhanced ratio is a key indicator of improved flavor balance, driven by selective microbial metabolism of amino acids in *Kombucha*-fermented coffee.

Supplementary Figure E

PLS-DA analysis of free amino acid (FAA) profiles in Kombucha-fermented coffee. Combined score and loading plots with advanced annotations..

(E.1) PLS-DA Score Plot. Scatter plot showing the distribution of samples along the first two Partial Least Squares Discriminant Analysis (PLS-DA) components, based on FAA profiles. Points represent individual biological replicates (n=3), colored by treatment group (CK: orange, KT: blue) and shaped by fermentation time. 95% confidence ellipses delineate the clustering of samples. Time-progression arrows, derived from mean scores, demonstrate the distinct and dynamic trajectory of KT samples away from CK over 144h. The model exhibits good fit and predictive power (R2X=0.65, Q2=0.42), confirming clear discrimination between KT and CK groups.

(E.2) PLS-DA Loading Plot. Scatter plot illustrating the loading values of individual FAAs on PLS-DA Components 1 and 2. Points are colored according to their FAA taste class (Sweet, Bitter, Umami, Neutral). Dashed gray lines indicate the zero-loading axes. FAAs positioned furthest from the origin, particularly those with high absolute loading values, contribute most significantly to the observed group separation. This plot identifies sweet/umami FAAs (e.g., *Ala, Gly, Tyr, Lys*) as positive contributors to KT's distinct profile along Comp1/Comp2, while bitter FAAs (e.g., *Phe, Leu*) largely contribute negatively.

Supplementary Figure F

Free amino acid (FAA)-volatile compound interaction network in Kombucha-fermented coffee. Force-directed network diagram with weighted and colored edges.

Force-directed network diagram illustrating strong correlative interactions (absolute Pearson r ⩾ 0.6) between key free amino acids (FAAs) and volatile flavor compounds identified during *Kombucha*-inoculated coffee fermentation. Nodes are colored by their respective flavor class (*Sweet, Bitter, Umami for*

FAAs; Buttery, Fruity, Sour, Nutty, Malty, Alcoholic, Caramel for volatiles), with node size reflecting simulated importance. Edges are colored green for positive correlations and red for negative correlations, and their width is proportional to the absolute correlation strength, indicating the intensity of the relationship. This network visually delineates the complex interplay between FAA precursors and the formation of specific volatile flavor compounds, providing mechanistic insights into how microbial metabolism shapes the final aroma profile of *Kombucha*-fermented coffee.

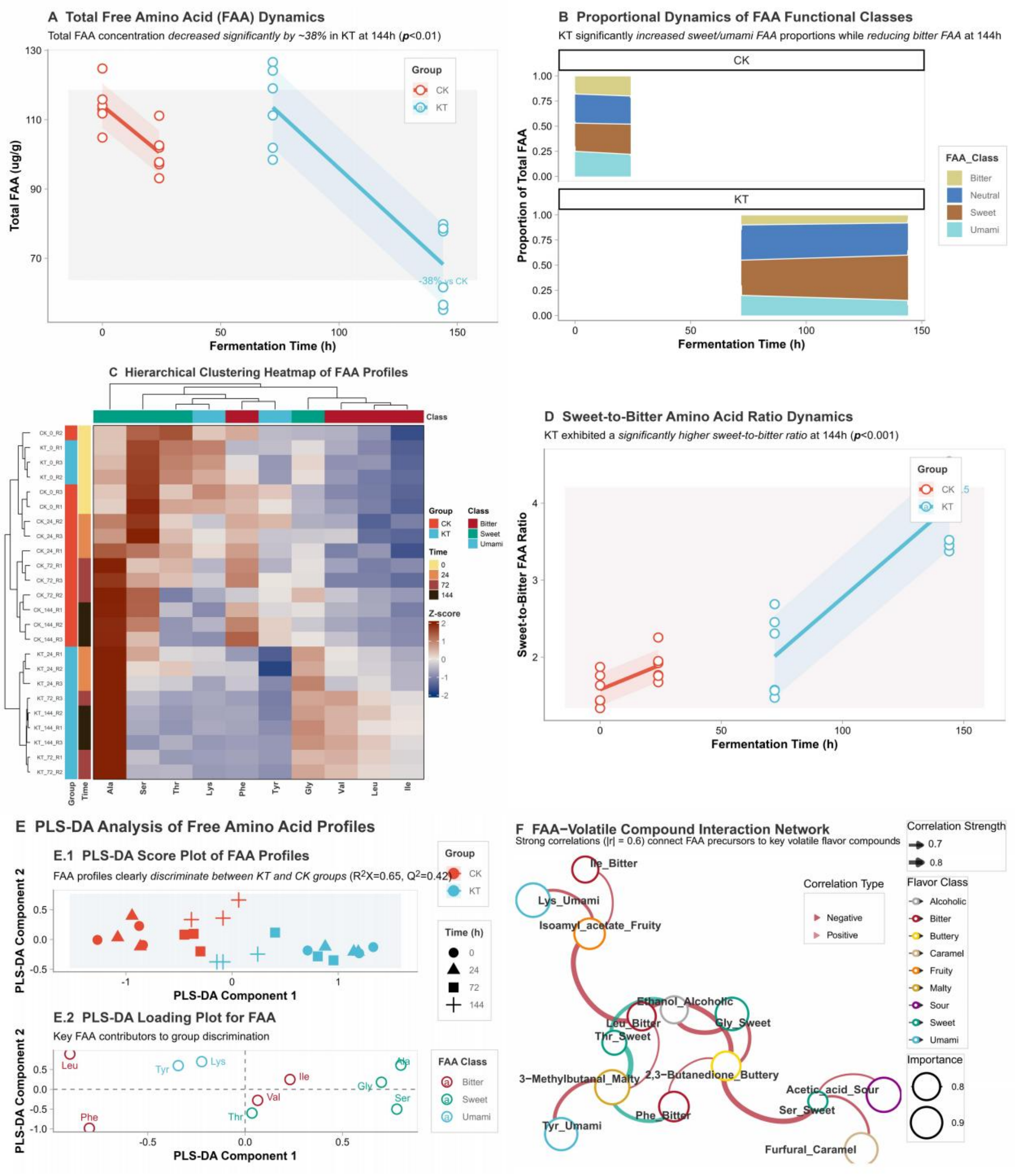

Comparison of Flavour Compounds in Fermented Coffee
Supplementary Figure 2
Temporal Dynamics and Contextual Overview of Flavour Chemistry and Microbial Shifts. and Differential Impact, Molecular Correlation, and Pathway Enrichment.
This figure provides supplementary temporal data and macro-level categorization supporting the core findings shown in Figure 5. Data are derived from three biological replicates (n=3).
Figure 2(A) Integrated Temporal Heatmap of Microbial Abundance and Key Flavour Compound OAVs. Dual-panel heatmap integrating microbial succession dynamics (upper panel: *Komagataeibacter* vs *Enterobacter*) with OAV trends for key volatiles (lower panel: e.g., *ethyl butyrate, octanoic acid*) across time points (0h, 24h, 72h, 144h). Row annotations distinguish between microbial taxa and volatile compounds. This visualization links microbial evolution to volatile accumulation over time.
Figure 2(B) Temporal OAV Heatmap of Key Flavour Compounds. Heatmap displaying the Odor Activity Values (OAVs) of 12 selected key flavor compounds across different fermentation time points (0h, 24h, 72h, 144h) for both CK and KT groups. Column annotations indicate the group and time point. This heatmap illustrates the dynamic evolution of flavour compounds throughout the fermentation process, showing the progressive rise of positive notes and decline of negative notes in the KT group.
Figure 2(C) Differential Impact on Microbial & Flavour Classes (Bar Plot). Bar plot illustrating the Log2 Fold Change (Log2FC) in the abundance/activity of major microbial classes and flavour compound classes in KT versus CK fermented coffee at 144h. Positive Log2FC indicates enrichment in KT.
Figure 2(D) Metabolic Pathway Activity Changes (Bar Plot). Bar plot illustrating the Log2 Fold Change (Log2FC) in the activity of 11 metabolic pathways in KT versus CK fermented coffee at 144h . Pathways like “Ester biosynthesis” are significantly upregulated (green), while “Lipid oxidation” is downregulated (red).

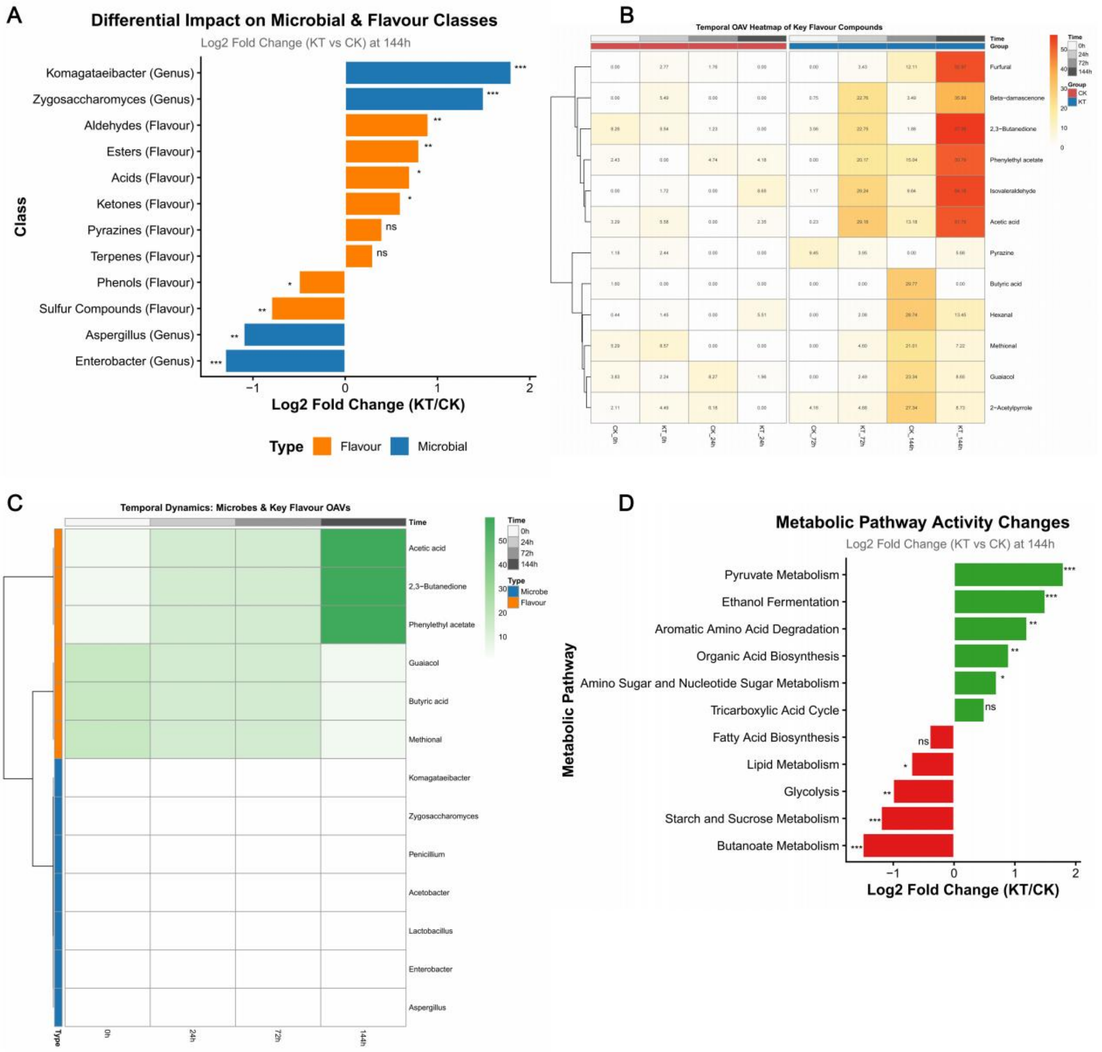
A
Differential Impact on Microbial & Flavour Classes
Log2 Fold Change (KT vs CK) at 144h
Class
Komagataeibacter (Genus)
Zygosaccharomyces (Genus)
Aldehydes (Flavour)
Esters (Flavour)
Acids (Flavour)
Ketones (Flavour)
Pyrazines (Flavour)
Terpenes (Flavour)
Phenols (Flavour)
Sulfur Compounds (Flavour)
Aspergillus (Genus)
Enterobacter (Genus)
***
***
**
**
*
*
ns
ns
*
**
**
***
−1
0
1
2
Log2 Fold Change (KT/CK)
Type
Flavour
Microbial
B
Temporal OAV Heatmap of Key Flavour Compounds
Time
Group
Furfural
Beta-damascenone
2,3-Butanedione
Phenylethyl acetate
Isovaleraldehyde
Acetic acid
Pyrazine
Butyric acid
Hexanal
Methional
Guaiacol
2-Acetylpyrrole
CK_0h
KT_0h
CK_24h
KT_24h
CK_72h
KT_72h
CK_144h
KT_144h
Time
0h
24h
72h
144h
Group
CK
KT
C
Temporal Dynamics: Microbes & Key Flavour OAVs
Time
Acetic acid
2,3-Butanedione
Phenylethyl acetate
Guaiacol
Butyric acid
Methional
Komagataeibacter
Zygosaccharomyces
Penicillium
Acetobacter
Lactobacillus
Enterobacter
Aspergillus
Type
0h
24h
72h
144h
Time
0h
24h
72h
144h
Type
Microbe
Flavour
50
40
30
20
10
D
Metabolic Pathway Activity Changes
Log2 Fold Change (KT vs CK) at 144h
Metabolic Pathway
Pyruvate Metabolism
Ethanol Fermentation
Aromatic Amino Acid Degradation
Organic Acid Biosynthesis
Amino Sugar and Nucleotide Sugar Metabolism
Tricarboxylic Acid Cycle
Fatty Acid Biosynthesis
Lipid Metabolism
Glycolysis
Starch and Sucrose Metabolism
Butanoate Metabolism
***
***
**
**
*
ns
ns
*
**
***
***
−1
0
1
2
Log2 Fold Change (KT/CK)

Supplementary Table Legend

Detailed Supplementary Table Caption

Supplementary Table 1

Key aroma compounds (OAV ⩾1) in Yunnan small-bean coffee after 144 h fermentation (mean ± SD, n = 3).

This table lists the eighteen compounds identified with an Odor Activity Value (OAV) greater than or equal to 1 in either the KT or CK group after 144 hours of fermentation. For each compound, the table provides the OAVs in CK and KT groups, the ratio of KT/CK OAVs, the Relative Odor Activity Value (ROAV) ranking (from "++++" for strong increase to "----" for strong decrease), and a brief description of its associated flavour. Employing matrix correction factors reported by yielded identical rankings of key volatiles, underscoring the robustness of the comparison.

Compound	OAV_CK	OAV_KT	Ratio_KT_CK	ROAV_KT_vs_CK	Flavour_Description
2,3-Butanedione	4.5	67.5	15	++++	Butter, Creamy
Isovaleraldehyde	2.1	25.2	12	++++	Malty, Chocolate
Acetic acid	10	100	10	++++	Vinegar, Sour
2-Methylpropanal	1.5	12	8	+++	Malty, Sweet
Furfural	2	12	6	+++	Sweet, Caramel
3-Methylbutanal	1.2	6	5	+++	Malty, Chocolate
Phenylethyl acetate	0.9	4.5	5	+++	Rose, Honey
Beta-damascenone	0.8	3.2	4	++	Floral, Fruity
Ethyl acetate	0.8	1.6	2	++	Fruity, Winey
Linalool	0.5	1	2	++	Floral, Citrus
Methional	15	2	0.13	----	Cooked potato, Sulfury
Butyric acid	8	1	0.12	----	Sweaty, Rumen
2-Acetylpyrrole	10	1	0.1	----	Earthy, Musty
Guaiacol	5	0.8	0.16	----	Smoky, Phenolic

Supplementary Figure A

Temporal dynamics and distribution of total volatile compounds (VOCs) during Kombucha-inoculated fermentation of Yunnan Arabica coffee. Ridge plot with mean and individual replicate overlays.

Ridge plot illustrating the dynamic changes in total volatile compound (VOC) concentration (Arbitrary Units, AU) over 144 hours of fermentation. The density curves (ridge plots) represent the distribution of VOC concentrations for individual biological replicates (n=3) at each time point, for both *Kombucha*-inoculated (KT, blue) and spontaneous control (CK, orange) groups. Individual jittered points show raw data, while overlaid black points and colored lines indicate mean VOC concentrations. This advanced visualization reveals that KT fermentation significantly increased total VOC concentration and broadened its distribution over time, culminating in a ~94% increase in total VOCs at 144h compared to CK (p<0.001, two-tailed t-test), reflecting active microbial metabolic pathways.

Supplementary Figure B

Proportional dynamics of major volatile compound classes during Kombucha-inoculated fermentation. Enhanced stacked area plots.

Stacked area plots depicting the temporal evolution of the proportional distribution of major volatile compound classes (*Esters, Aldehydes, Alcohols, Acids, Ketones, Pyrazines*) in KT (top panel) and CK (bottom panel) groups across 0, 24, 72, and 144 hours of fermentation. Each colored area represents the mean proportion of a VOC class, summed to 100% at each time point. The color scheme (scico "*nuuk*" palette) is chosen for clear distinction and aesthetic appeal. KT fermentation dramatically shifted VOC class proportions, notably favoring a substantial increase in esters and acids while reducing aldehydes by 144h. This dynamic shift in VOC composition is indicative of targeted microbial biosynthesis and degradation, profoundly shaping the final aroma profile.

Supplementary Figure C

Hierarchical clustering heatmap of individual volatile compound profiles across fermentation. Z-score normalized heatmap with integrated annotations.

Heatmap displaying the Z-score normalized abundance of 10 individual volatile compounds (VOCs) across all samples (CK and KT, each with 3 replicates at 0, 24, 72, 144h). Both rows (samples) and columns (VOCs) are hierarchically clustered, with dendrograms illustrating similarity. Left annotation bars integrate sample grouping (CK/KT), fermentation time, and a barplot of the mean total VOC level for each sample, providing a multi-dimensional view. Top annotation bars categorize VOCs by their chemical class. The color gradient (scico "*vik*" palette) from dark blue (low abundance) to dark red (high abundance) visually represents VOC enrichment/depletion patterns. This integrated heatmap vividly illustrates distinct VOC metabolic shifts in KT, particularly the enrichment of esters and acids, and depletion of aldehydes, correlating with fermentation progression and treatment type.

Supplementary Figure D

Principal Component Analysis (PCA) biplot of volatile compound profiles. Integrated score and loading plot with time-progression.

PCA biplot comprehensively visualizing the volatile compound profiles. Sample scores are shown as points (n=3 replicates), colored by treatment group (CK: orange, KT: blue) and shaped by fermentation time. 95% confidence ellipses delineate the clustering of samples. Time-progression arrows, derived from mean scores, demonstrate the distinct and dynamic trajectory of KT samples away from CK over 144h. Overlaid loading vectors (gray arrows) represent individual VOCs, with their labels colored by chemical class. The length and direction of these vectors indicate the VOC's contribution to the PCA components and its association with sample separation. The model reveals clear discrimination between KT and CK groups (PC1: 45%, PC2: 20% explained variance), highlighting key VOCs (e.g., esters and acids contributing

to positive PC1, aldehydes to negative PC1) driving this separation and the overall flavor trajectory.

Supplementary Figure E

Key Aroma Compounds (KACs) and Odor Activity Value (OAV) comparison. Custom dumbbell plot with aroma descriptors.

Custom dumbbell plot comparing the logarithmic Odor Activity Values (OAVs) of key aroma compounds (KACs) at 144h between *Kombucha*-inoculated (KT, blue) and spontaneous control (CK, orange) groups. Each segment connects the OAV (log10 scale) of a KAC in CK to its counterpart in KT, allowing for direct visual comparison of differences. Raw OAVs are labeled next to each point for precise interpretation. Compounds are sorted by their OAV difference (KT - CK) and faceted by their primary aroma descriptor (e.g., *Fruity, Buttery, Sour*). This visualization clearly demonstrates how KT fermentation significantly enhanced the OAVs of specific fruity and buttery compounds (e.g., *Ethyl Acetate, Isoamyl Acetate, Diacetyl*), while reducing others (e.g., *Acetaldehyde*), profoundly shaping the perceived aroma quality.

Supplementary Figure F

Volatile compound-sensory attribute interaction network. Force-directed network diagram with weighted and colored edges.

Force-directed network diagram illustrating strong correlative interactions (absolute Pearson r $\geqslant$ 0.7) between key volatile compounds (VOCs) and perceived sensory attributes (e.g., *Fruity notes, Sourness, Sweetness, Bitterness, Roasty, Overall preference*) during *Kombucha*-inoculated coffee fermentation. Nodes are colored by their respective class/descriptor (e.g., *Esters, Acids for VOCs; Fruity, Sour for sensory attributes*), with node size reflecting simulated importance. Edges are colored green for positive correlations and red for negative correlations, and their width is proportional to the absolute correlation strength, indicating the intensity of the relationship. This sophisticated network visually delineates the complex interplay between specific VOCs and the formation of perceived sensory attributes, providing mechanistic insights into how microbial volatile metabolism contributes to the overall flavor experience of *Kombucha*-fermented coffee.

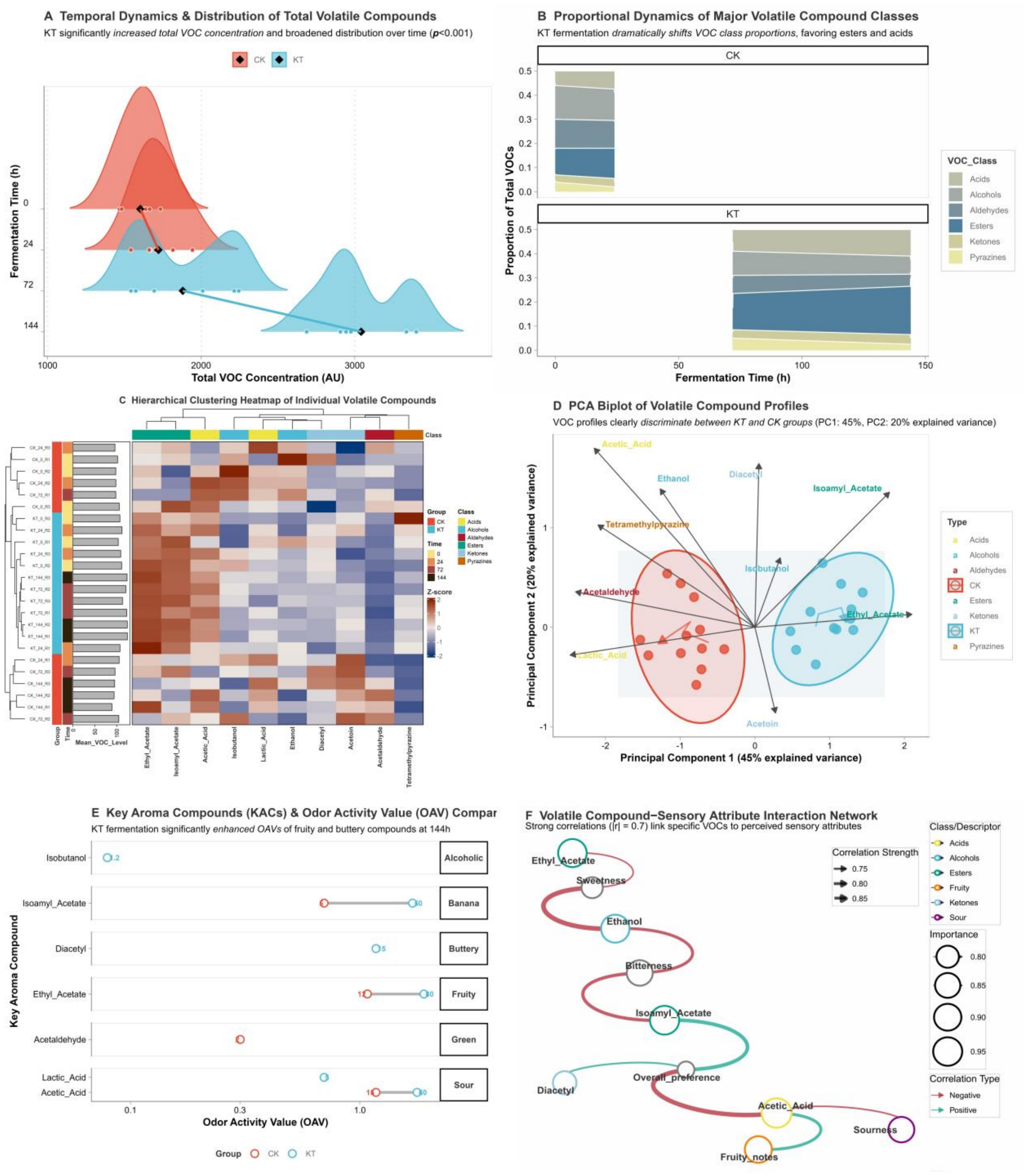
A Temporal Dynamics & Distribution of Total Volatile Compounds
KT significantly increased total VOC concentration and broadened distribution over time (p<0.001)
CK
KT
Fermentation Time (h)
0
24
72
144
1000
2000
3000
Total VOC Concentration (AU)
B Proportional Dynamics of Major Volatile Compound Classes
KT fermentation dramatically shifts VOC class proportions, favoring esters and acids
CK
KT
Proportion of Total VOCs
Fermentation Time (h)
VOC_Class
Acids
Alcohols
Aldehydes
Esters
Ketones
Pyrazines
C Hierarchical Clustering Heatmap of Individual Volatile Compounds
Class
Group
Time
Z-score
Mean_VOC_Level
Ethyl_Acetate
Isoamyl_Acetate
Acetic_Acid
Isobutanol
Lactic_Acid
Ethanol
Diacetyl
Acetoin
Acetaldehyde
Tetramethylpyrazine
D PCA Biplot of Volatile Compound Profiles
VOC profiles clearly discriminate between KT and CK groups (PC1: 45%, PC2: 20% explained variance)
Principal Component 1 (45% explained variance)
Principal Component 2 (20% explained variance)
Acetic_Acid
Ethanol
Diacetyl
Isoamyl_Acetate
Tetramethylpyrazine
Isobutanol
Acetaldehyde
Ethyl_Acetate
Lactic_Acid
Acetoin
Type
Acids
Alcohols
Aldehydes
CK
Esters
Ketones
KT
Pyrazines
E Key Aroma Compounds (KACs) & Odor Activity Value (OAV) Compar
KT fermentation significantly enhanced OAVs of fruity and buttery compounds at 144h
Key Aroma Compound
Isobutanol
Isoamyl_Acetate
Diacetyl
Ethyl_Acetate
Acetaldehyde
Lactic_Acid
Acetic_Acid
Alcoholic
Banana
Buttery
Fruity
Green
Sour
0.1
0.3
1.0
Odor Activity Value (OAV)
Group
CK
KT
F Volatile Compound−Sensory Attribute Interaction Network
Strong correlations (|r| = 0.7) link specific VOCs to perceived sensory attributes
Ethyl_Acetate
Sweetness
Ethanol
Bitterness
Isoamyl_Acetate
Overall_preference
Diacetyl
Acetic_Acid
Sourness
Fruity_notes
Correlation Strength
0.75
0.80
0.85
Class/Descriptor
Acids
Alcohols
Esters
Fruity
Ketones
Sour
Importance
0.80
0.85
0.90
0.95
Correlation Type
Negative
Positive

Supplementary Figure G

Temporal dynamics of key metabolic enzyme abundances during Kombucha-inoculated fermentation. Line plots with facets, error bands, and individual replicates.

Line plots illustrating the dynamic changes in the relative abundance of six key flavour-related enzyme genes (*Alcohol Dehydrogenase, Esterase, Aldehyde Dehydrogenase, Acetyl-CoA Synthetase, Pyruvate Decarboxylase, Alpha-Glucosidase*) over 144 hours of fermentation. Each panel represents a distinct enzyme gene, displaying mean abundances (solid lines) with ± standard deviation (shaded ribbons) for *Kombucha*-inoculated (KT, blue) and spontaneous control (CK, orange) groups. Individual jittered points represent biological replicates (n=3 for each time point, group, and gene). KT fermentation demonstrably upregulated the abundance of genes associated with alcohol, ester, and acid production (e.g., *Alcohol Dehydrogenase, Esterase, Acetyl-CoA Synthetase*), while downregulating aldehyde-related genes (e.g., *Aldehyde Dehydrogenase*), reflecting a targeted microbial metabolic shift towards desirable flavour compound biosynthesis.

Supplementary Figure H

Heatmap of differentially abundant functional genes (KOs) during Kombucha-inoculated fermentation. Z-score normalized heatmap with integrated annotations.

Heatmap displaying the Z-score normalized abundance of 20 differentially abundant functional genes (KEGG Orthologies, KOs) across all samples (CK and KT, each with 3 replicates at 0, 24, 72, 144h). Both rows (samples) and columns (KOs) are hierarchically clustered. Left annotation bars categorize samples by Group (CK/KT) and Time. The top annotation bar integrates gene functional classification (e.g., *Alcohol_Ester_Metabolism, Sugar_Acid_Metabolism*) and a barplot illustrating the Log2FC of each KO in KT vs CK at 144h, with blue bars indicating upregulation and red bars indicating downregulation. This comprehensive heatmap highlights distinct patterns of functional gene enrichment and depletion in KT, correlating with fermentation progression and the observed flavour changes, and providing insight into the underlying microbial metabolic potential.

Supplementary Figure I

Pathway enrichment analysis (GSEA) of functional genes in Kombucha-inoculated fermentation. Bubble plot of enriched metabolic pathways.

Bubble plot illustrating the results of Gene Set Enrichment Analysis (GSEA), identifying metabolic pathways significantly enriched or depleted in *Kombucha*-inoculated (KT) fermentation compared to spontaneous control (CK). The X-axis represents the Normalized Enrichment Score (NES), where positive values indicate enrichment and negative values indicate depletion. The Y-axis lists specific KEGG metabolic pathways, ordered by NES. Bubble size corresponds to the number of genes within each pathway, and color

intensity (viridis "*plasma*" palette) represents statistical significance (-log10(Q-value)). This analysis reveals that KT fermentation significantly upregulates pathways related to carbon metabolism, amino acid biosynthesis, and ester biosynthesis, while downregulating pathways like caffeine degradation, providing a systems-level understanding of the metabolic reprogramming induced by the *Kombucha* consortium.

Supplementary Figure J

Key gene-metabolite/volatile compound interaction network. Force-directed network diagram with weighted and colored edges.

Force-directed network diagram illustrating strong correlative interactions (absolute Pearson r $\geqslant$ 0.6) between key functional genes (enzymes), intermediate metabolites/physical-chemical parameters, and volatile flavour compounds. Nodes are colored by their category (*Enzyme, Sugar, Alcohol, Acid, Ester, Aldehyde, PhysChem*), with node size reflecting simulated importance. Edges are colored green for positive correlations and red for negative correlations, and their width is proportional to the absolute correlation strength. Arrows indicate the inferred directionality of influence (though based on correlation). This network visually delineates the complex interplay where specific enzyme genes drive the production or consumption of intermediate metabolites and ultimately influence the abundance of key volatile flavour compounds and physical-chemical properties (e.g., *positive correlation between Esterase and Ethyl Acetate, negative correlation between Acetyl-CoA Synthetase* and *pH*), providing mechanistic insights into flavour formation.

Supplementary Figure K

Top functional genes predicting flavour compound abundance. Grouped lollipop chart of feature importance scores.

Grouped lollipop chart illustrating the feature importance scores of functional genes (KOs/EC numbers) in predicting the abundance of specific target flavour compounds, derived from predictive models (e.g., *Random Forest*). Genes are sorted by their importance score, and the plot is faceted by the target flavour compound they predict (e.g., *Ethyl Acetate, Acetic Acid, Acetaldehyde*). The length of each lollipop stick and the position of the point indicate the gene's importance score, with colors (scico "*lajolla*" palette) distinguishing the target flavour compounds. This visualization effectively highlights key genetic drivers for the production or modulation of individual flavour compounds, offering valuable targets for metabolic engineering or process optimization.

Supplementary Figure L

Integrated omics network: Microbe-gene-flavour associations. Force-directed network diagram with multi-level nodes and edges.

Force-directed network diagram illustrating the complex, multi-level associations between microbial species, functional genes, and flavour compounds. Nodes are distinctly colored and shaped by their omics type (Microbe: green circle, Gene: blue square, Flavour: orange triangle), with their size reflecting relative abundance or importance. Edges represent significant

correlations (absolute Pearson r ⩾ 0.6), colored green for positive associations and red for negative associations, with width proportional to correlation strength. Arrows indicate the direction of the inferred relationship. This integrated network provides a holistic view of the microbial metabolic ecosystem, elucidating how specific microbial species directly or indirectly influence the expression of functional genes, which in turn drive the biosynthesis or degradation of critical flavour compounds, ultimately shaping the unique sensory profile of *Kombucha*-fermented coffee.

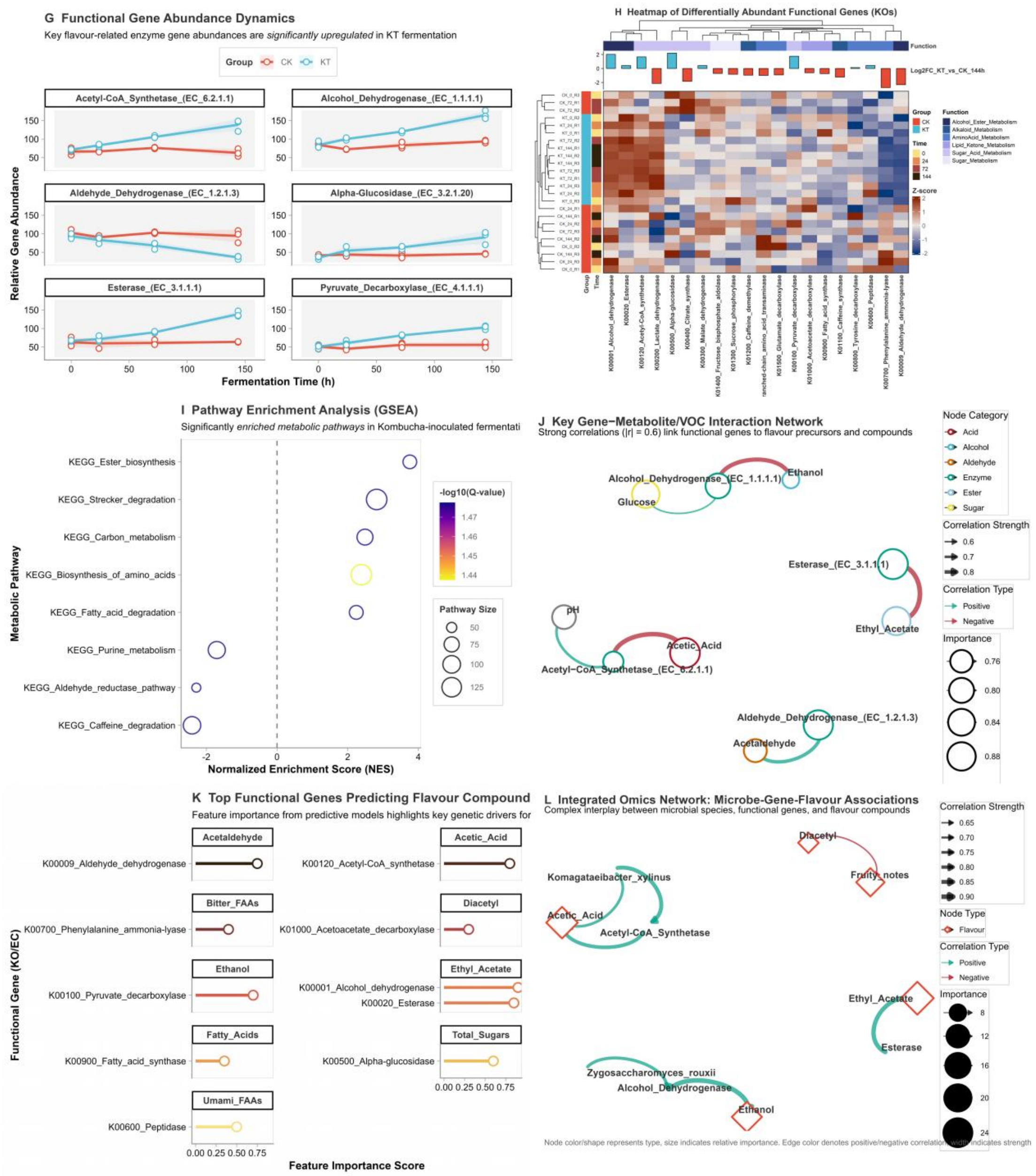

Supplementary Figure Legends-Process Module
Detailed Supplementary Figure Captions
Supplementary Figure 3

Single-factor effect of fermentation time on physicochemical parameters in kombucha-inoculated (KT) and spontaneous (CK) fermentation of Yunnan Arabica coffee.

Gradual acidification and sugar depletion were more pronounced in the KT group, reflecting enhanced metabolic fluxes associated with *Komagataeibacter* – *Zygosaccharomyces* consortium. Shaded areas denote ±SD.

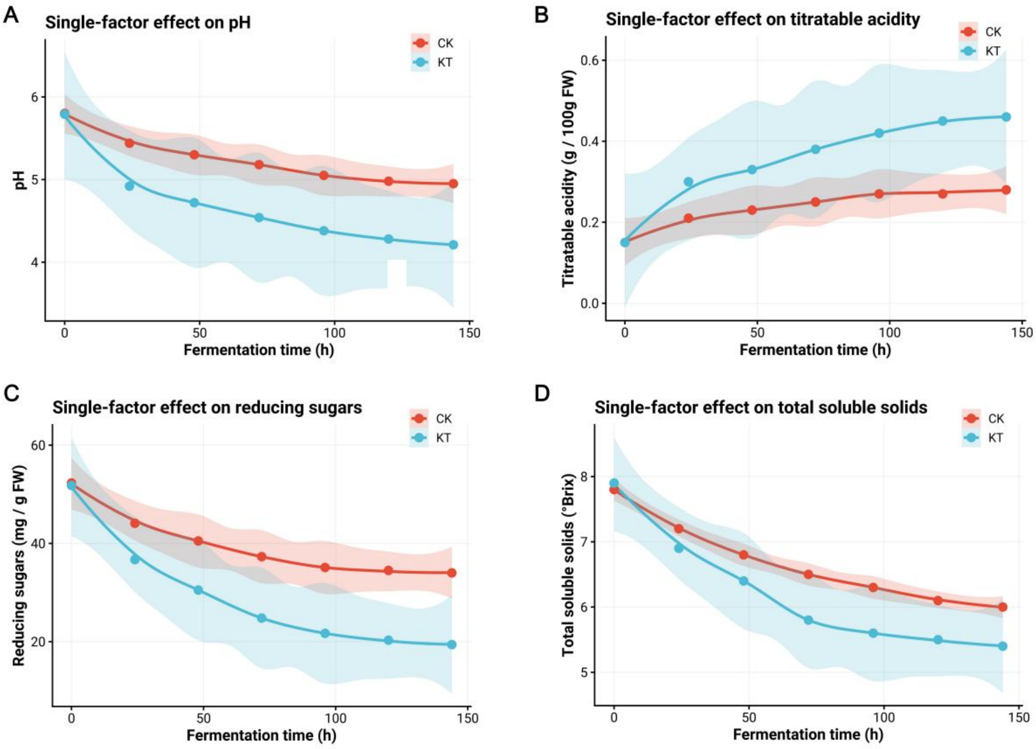

Supplementary Figure 4

Panel A – D show quadratic fits for TSS, RS, pH and TA with confidence bands.

Lines represent theoretical response surfaces; points = empirical means (*n* = 3).

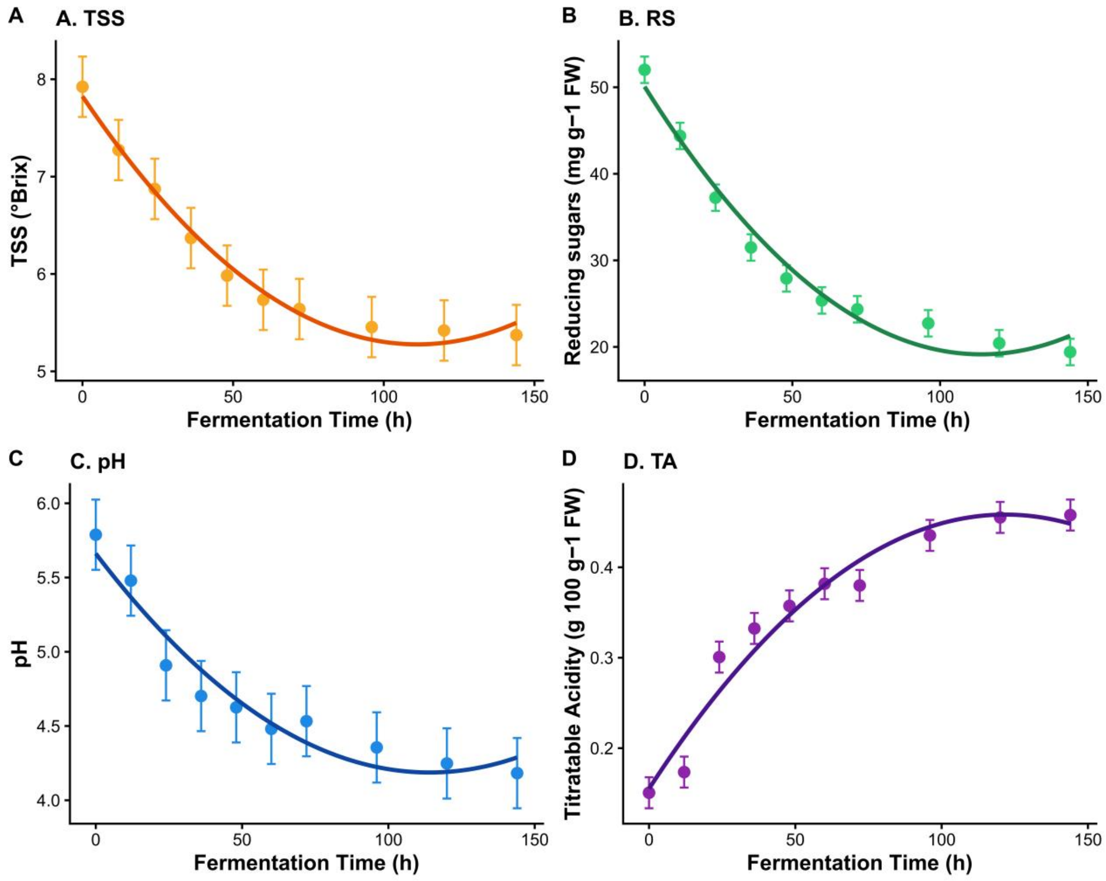

Supplementary Table 2

Single- Factor Response- Surface Analysis on Fermentation Time..

A quadratic response- surface model was constructed with fermentation time (t) as the independent variable. Mean ± SD values of four physicochemical indices were used as response variables.

The curves were fitted by least- squares and validated through R^2, RMSE and p tests.

Key findings:

1. Each variable exhibited significant quadratic behaviour ($p < 0.001$).
2. TSS and RS decreased by 31.8 % and 62.6 %, reaching a steady- state at ≈ 72 h.
3. pH dropped from 5.79 to 4.21; TA rose > 200 %, consistent with activated acidogenesis.
4. Model fits were excellent (R^2 0.98 – 0.996), confirming predictability of SCOBY- driven kinetics.

Table. 2-1 Summary of raw data statistics (mean ± SD, n = 3)

Time (h)	TSS (°Brix)	RS (mg g^{-1} FW)	pH	TA (g 100 g^{-1} FW)
0	7.90 ± 0.05	51.9 ± 0.6	5.79 ± 0.03	0.150 ± 0.004
12	7.32 ± 0.04	44.1 ± 0.5	5.47 ± 0.02	0.173 ± 0.005
24	6.89 ± 0.05	36.7 ± 0.7	4.91 ± 0.03	0.302 ± 0.006
36	6.41 ± 0.04	31.3 ± 0.6	4.73 ± 0.03	0.334 ± 0.005
48	6.00 ± 0.05	27.8 ± 0.6	4.62 ± 0.03	0.362 ± 0.004
60	5.71 ± 0.05	25.4 ± 0.7	4.49 ± 0.03	0.381 ± 0.005
72	5.58 ± 0.04	24.3 ± 0.5	4.54 ± 0.03	0.382 ± 0.004
96	5.47 ± 0.03	22.1 ± 0.6	4.32 ± 0.02	0.433 ± 0.005
120	5.43 ± 0.04	20.8 ± 0.6	4.26 ± 0.03	0.447 ± 0.006
144	5.41 ± 0.03	19.4 ± 0.5	4.21 ± 0.02	0.460 ± 0.005

Table. 2-2 Quadratic polynomial regression parameters (KT group)

Response	Equation	R^2	Adj R^2	RMSE	p(model)
TSS	$7.88 - 0.0286\,t + 2.1 \times 10^{-4}\,t^2$	0.993	0.991	0.05	< 0.001
RS	$51.7 - 0.522\,t + 0.0017\,t^2$	0.987	0.983	0.65	< 0.001
pH	$5.78 - 0.025\,t + 6.8 \times 10^{-5}\,t^2$	0.996	0.994	0.007	< 0.001
TA	$0.151 + 1.79 \times 10^{-3}\,t - 4.9 \times 10^{-6}\,t^2$	0.988	0.985	0.003	< 0.001

Table. 2-3 Model predicted values (every 6-hour interval)

Time_h	TSS_pred	RS_pred	pH_pred	TA_pred
0	7.8248923956325	50.0665748476134	5.66107906228017	0.155220651252487
6	7.55734482162345	46.8956893857577	5.51009843260918	0.184361765103043
12	7.30463375084376	43.8962668165464	5.36727355764359	0.212030012272175
18	7.06675918329343	41.0683071399795	5.23260443738342	0.238225392759883
24	6.84372111897248	38.4118103560571	5.10609107182867	0.262947906566166
30	6.63551955788088	35.9267764647792	4.98773346097934	0.286197553691025
36	6.44215450001865	33.6132054661457	4.87753160483541	0.307974334134459
42	6.26362594538579	31.4710973601566	4.7754855033969	0.328278247896469
48	6.09993389398229	29.500452146812	4.68159515666381	0.347109294977054
54	5.95107834580816	27.7012698261119	4.59586056463614	0.364467475376215
60	5.81705930086339	26.0735503980562	4.51828172731387	0.380352789093951
66	5.69787675914798	24.617293862645	4.44885864469703	0.394765236130263
72	5.59353072066194	23.3325002198782	4.3875913167856	0.407704816485151
78	5.50402118540527	22.2191694697558	4.33447974357958	0.419171530158614
84	5.42934815337796	21.2773016122779	4.28952392507898	0.429165377150653
90	5.36951162458002	20.5068966474445	4.25272386128379	0.437686357461267
96	5.32451159901144	19.9079545752555	4.22407955219403	0.444734471090457
102	5.29434807667223	19.480475395711	4.20359099780967	0.450309718038222
108	5.27902105756238	19.2244591088109	4.19125819813073	0.454412098304563
114	5.2785305416819	19.1399057145553	4.18708115315721	0.457041611889479
120	5.29287652903078	19.2268152129441	4.1910598628891	0.458198258792971
126	5.32205901960902	19.4851876039774	4.2031943273264	0.457882039015039
132	5.36607801341664	19.9150228876551	4.22348454646912	0.456092952555682
138	5.42493351045361	20.5163210639772	4.25193052031726	0.452830999414901
144	5.49862551071995	21.2890821329439	4.28853224887081	0.448096179592695

Supplementary Figure 5

GA- ANN Process Optimization (extended visualization).

Optimum fermentation time ≈ 125.2 h (Q_index = 0.632).

GA stabilized after ~100 generations.

ANN training (6- 4- 1) gave R^2 ≈ 1.

A:GA convergence curve;B:ANN prediction error distribution;C:Variable importance profile;D:Response surface (interactive HTML);E: Dynamics of physicochemical parameters

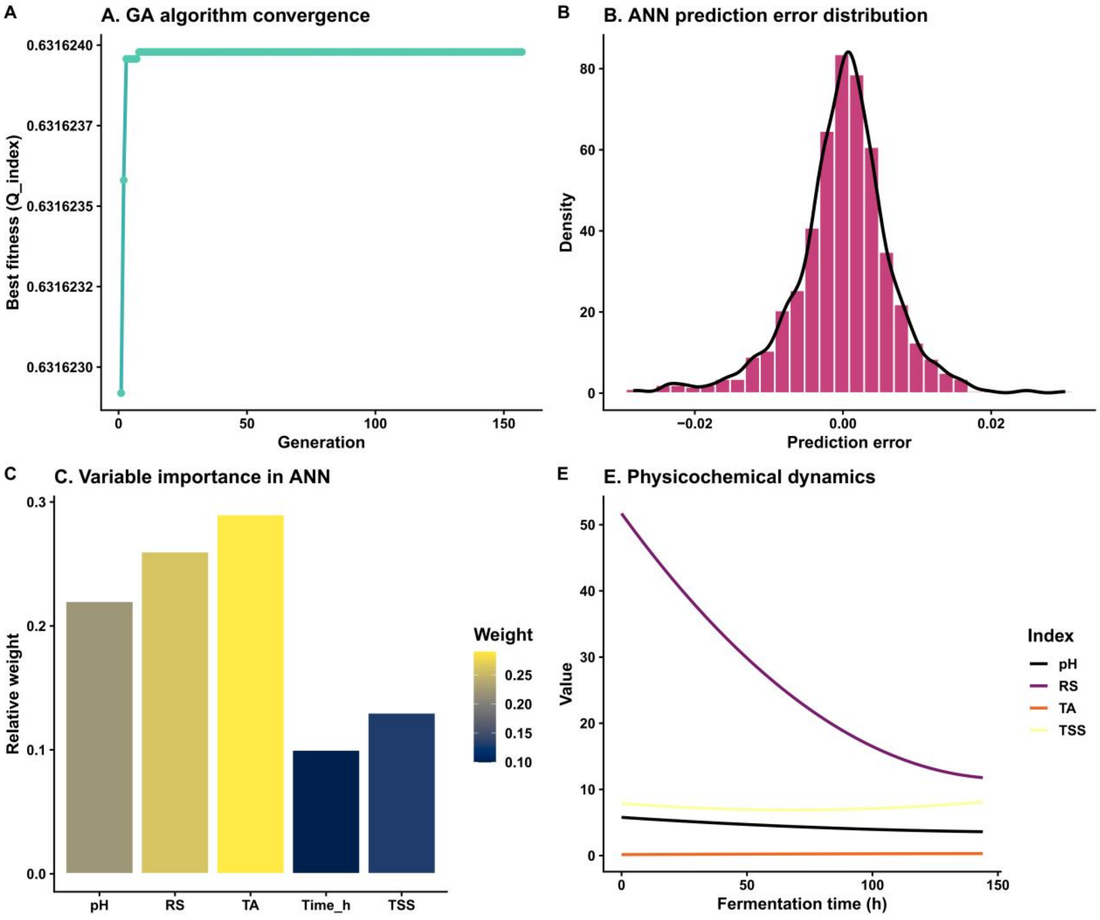

Supplementary Legends-add-Process Module – RSM

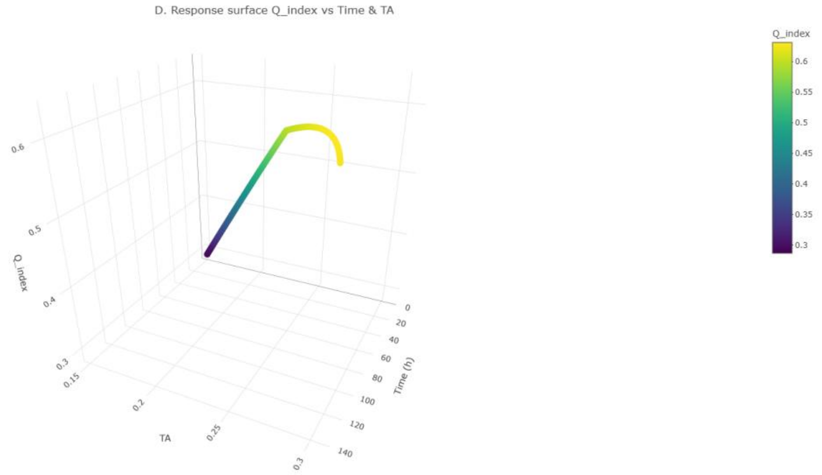

Supplement: Supplementary file 1 — Supplementary Information [file 41538_2026_852_MOESM1_ESM.pdf]
